# Novel neoadjuvant therapies for muscle‐invasive bladder cancer: Systematic review and meta‐analysis

**DOI:** 10.1002/bco2.70031

**Published:** 2025-05-26

**Authors:** Shugo Yajima, Naoki Imasato, Hitoshi Masuda

**Affiliations:** ^1^ Department of Urology National Cancer Center Hospital East Chiba Japan

**Keywords:** immunotherapy, meta‐analysis, molecular targeted therapy, neoadjuvant therapy, urinary bladder neoplasms

## Abstract

**Objectives:**

This study aims to evaluate the efficacy and safety of novel neoadjuvant therapies including immune checkpoint inhibitors (ICI), molecular targeted agents (MTA) and antibody‐drug conjugates in muscle‐invasive bladder cancer through a systematic review and meta‐analysis of prospective clinical trials.

**Subjects/Patients and Methods:**

A systematic search was performed using PubMed, Web of Science, Cochrane Library, Google Scholar and ClinicalTrials.gov through December 2024. Eligible studies were phase II or higher prospective trials investigating novel agents as neoadjuvant therapy. Primary endpoints were pathologic complete response and downstaging rates. Secondary endpoints included biomarker analysis, survival outcomes and safety profiles. Data were extracted following PRISMA guidelines, and random‐effects meta‐analyses were performed.

**Results:**

Seventeen trials comprising 1977 patients were analysed. ICI‐based treatments were associated with pathologic complete response rates of 37% (95% CI, 34%–40%) and downstaging rates of 55% (95% CI, 48%–61%). ICI monotherapy was associated with higher response rates compared with MTA monotherapy (pathologic complete response: 34% vs. 5%; downstaging: 47% vs. 27%). Grade ≥3 adverse events occurred less frequently with ICI‐based treatments than MTA‐based treatments (35% vs. 62%). High PD‐L1 expression was associated with improved pathologic response (OR, 2.60; 95% CI, 1.44–4.71). Two‐year overall survival rates were 81% for ICI‐based regimens and 86% for MTA‐based regimens.

**Conclusion:**

Novel neoadjuvant therapies, particularly ICI‐based regimens, were associated with meaningful pathologic response rates and acceptable safety profiles. PD‐L1 expression may help guide patient selection for ICI‐based therapy. Randomized trials are needed to establish optimal treatment algorithms and validate predictive biomarkers.

## 1 INTRODUCTION

For muscle‐invasive bladder cancer (MIBC), the standard of care has been neoadjuvant chemotherapy (NAC) followed by radical cystectomy when feasible.^1^ Traditional cisplatin‐based NAC has demonstrated pathological complete response (pCR) rates of approximately 30%^2^, ^3^, ^4^ and an absolute improvement in 5‐year overall survival of 5% in historical meta‐analyses.^5^ However, significant toxicity and limited response rates underscore the need for more effective therapeutic approaches.

The therapeutic landscape for neoadjuvant treatment has evolved substantially in recent years. The emergence of immune checkpoint inhibitors (ICIs), antibody‐drug conjugates (ADCs) and molecular targeted agents (MTAs) has expanded treatment options beyond conventional chemotherapy.^6^, ^7^ Recent clinical trials investigating the combination of ICIs with chemotherapy have reported pCR rates of approximately 50%, potentially surpassing historical outcomes with conventional chemotherapy.^8^

The advent of novel therapeutic approaches has prompted investigations into potential biomarker‐guided treatment selection. Although programmed death‐ligand 1 (PD‐L1) expression status and tumour mutational burden (TMB) have emerged as predictive markers for immunotherapy response in several cancer types,^9^ their predictive value for neoadjuvant immunotherapy in bladder cancer remains to be established. Despite expanding therapeutic options, the relative efficacy and optimal sequencing of various neoadjuvant approaches remain unclear, particularly across different molecular subtypes and patient populations, and there has not been a comprehensive analysis integrating their efficacy and safety outcomes. As treatment options continue to multiply, establishing the comparative effectiveness and safety profiles of these therapies becomes increasingly important for informed clinical decision‐making.

This systematic review and meta‐analysis aims to evaluate the outcomes of novel neoadjuvant therapies (ICIs, ADCs and MTAs) in patients with muscle‐invasive bladder cancer. Our analysis specifically focuses on both short‐term outcomes, including pCR rates, downstaging rates and surgical feasibility, as well as long‐term outcomes such as disease‐free and overall survival. Additionally, we conduct a detailed assessment of treatment‐related adverse events to provide a comprehensive understanding of the risk–benefit profiles of these therapeutic approaches.

## 2 MATERIALS AND METHODS

This systematic review and meta‐analysis was conducted according to the Preferred Reporting Items for Systematic Reviews and Meta‐Analyses (PRISMA) guidelines.^10^ The protocol of this study was preregistered in the International Prospective Register of Systematic Reviews database (PROSPERO: CRD42024626545). The PRISMA checklist 2020 is shown in Table S1.

### 2.1 Literature search strategy

We systematically searched four electronic databases (PubMed, Web of Science, Cochrane Library and Google Scholar) and ClinicalTrials.gov from inception through December 2024. We also reviewed abstracts from major oncology conferences, specifically the European Society for Medical Oncology (ESMO) congress and the American Society of Clinical Oncology (ASCO) annual meeting. The search strategy included terms related to bladder cancer, neoadjuvant therapy, immunotherapy, molecular targeted agents, ADC and clinical trials. We also manually reviewed the reference lists of included articles. The complete search strategy is provided in Appendix S1.

### 2.2 Study selection

Inclusion criteria were as follows:

- Prospective clinical trials investigating neoadjuvant therapy in muscle‐invasive bladder cancer
- Studies investigating novel agents (including ICIs, ADCs and MTAs) either as monotherapy or in combination with chemotherapy
- Studies reporting pathological outcomes after radical cystectomy
- Studies that included concurrent radiotherapy or intravesical therapy were eligible if they met other inclusion criteria
- English language publications.

Exclusion criteria were as follows:

- Studies evaluating only conventional chemotherapy regimens without novel agents
- Studies without pathological outcome assessment
- Retrospective studies
- Case reports or series with <10 patients.

### 2.3 Outcomes

Primary outcomes were as follows:

- Pathological complete response rate (ypT0N0)
- Pathological downstaging rate (≤ypT1N0).

Secondary outcomes included the following:

- Treatment efficacy analysis in ICI‐treated patients stratified by biomarker status (PD‐L1 expression and TMB, both high versus low using trial‐defined cut‐offs) using pathological complete response (ypT0N0) or downstaging (≤ypT1N0) as endpoints
- 2‐year overall survival with 95% confidence intervals
- 2‐year recurrence‐free survival with 95% confidence intervals
- Grade ≥3 adverse events
- Specific adverse events of interest (e.g., immune‐related adverse events, anaemia, constipation, diarrhoea, fatigue, nausea, neutropenia and thrombocytopenia)
- Surgical resection rate (proportion of patients who proceeded to surgery)
- R0 resection rate.

### 2.4 Data extraction and quality assessment

Two investigators (S.Y. and N.I.) independently extracted data using standardized forms. In addition to clinical and pathological outcomes, we documented funding sources for each included study to enable transparent assessment of potential bias. For PD‐L1 expression analysis, we used the cut‐off values and scoring methods as defined in each original study, acknowledging the variations in PD‐L1 testing methods and criteria across studies. For randomized controlled trials (RCTs), we used the Cochrane Risk of Bias tool 2.0 (RoB 2).^11^ For non‐randomized studies, we used the Risk of Bias in Non‐randomized Studies of Interventions (ROBINS‐I) tool.^12^ Disagreements were resolved by consensus or third‐party adjudication. When data were unclear or missing in the published reports, we contacted the corresponding authors of the original studies for clarification. If no response was received, we used the available data.

### 2.5 Statistical analysis

All analyses were performed according to the intention‐to‐treat (ITT) principle using R version 4.3.1 (R Foundation for Statistical Computing, Vienna, Austria). For binary outcomes, we conducted random‐effects meta‐analyses using a generalized linear mixed model approach.^13^ For survival outcomes, we used restricted maximum likelihood estimation^14^ in the random‐effects model, incorporating survival rates and their 95% confidence intervals extracted from Kaplan–Meier analyses at 2 years.

Publication bias was assessed using Doi plots with LFK index.^15^ For studies reporting zero events, a continuity correction was applied by adding 0.5 to both the number of events and total sample size. The interpretation of LFK index was |LFK| ≤ 1 indicating no asymmetry, 1 < |LFK| ≤ 2 indicating minor asymmetry and |LFK| > 2 indicating major asymmetry.

Heterogeneity was assessed using *I* ^2^ statistics and Cochran's Q test, with *I* ^2^ values of <25%, 25%–75% and >75% indicating low, moderate and high heterogeneity, respectively. For trials with multiple treatment arms, each experimental arm was included separately in the analysis if the inclusion criteria were met.

Subgroup analyses were conducted according to the following:

- Treatment type (ICI monotherapy, ICI plus chemotherapy, MTA monotherapy, MTA plus chemotherapy)
- Biomarker status in ICI‐treated patients (PD‐L1 expression and TMB, both high versus low using trial‐defined cut‐offs).

For pCR and downstaging rates, we calculated proportions with exact 95% confidence intervals. For comparison of proportions between subgroups, we used Fisher's exact test for individual study comparisons and the Mantel–Haenszel method^16^ for meta‐analysis comparisons, generating odds ratios with 95% confidence intervals. For time‐to‐event outcomes, hazard ratios with 95% confidence intervals were calculated when possible. A sensitivity analysis was performed using only patients who underwent radical cystectomy as the denominator population, rather than the ITT population. This analysis aimed to evaluate whether the treatment effects differed when focusing specifically on patients who completed the planned surgical intervention.

All statistical tests were two‐sided, with *P* < 0.05 considered statistically significant. Analyses were performed using R version 4.3.1 with the metafor package^17^ for meta‐analyses.

## 3 RESULTS

### 3.1 Study selection and characteristics

Our initial literature search identified 939 records from PubMed (*n* = 285), Cochrane Library (*n* = 213), Web of Science (*n* = 269), Google Scholar (*n* = 58) and ClinicalTrials.gov (*n* = 114). After removing 140 duplicate records, 799 records remained for screening of titles and abstracts (Figure S1, PRISMA flow diagram). Following this screening, 768 articles were excluded, leaving 31 articles for full‐text review.^8^, ^18^, ^19^, ^20^, ^21^, ^22^, ^23^, ^24^, ^25^, ^26^, ^27^, ^28^, ^29^, ^30^, ^31^, ^32^, ^33^, ^34^, ^35^, ^36^, ^37^, ^38^, ^39^, ^40^, ^41^, ^42^, ^43^, ^44^, ^45^, ^46^, ^47^ Of these, 11 were excluded based on our inclusion criteria (six studies with insufficient data,^37^, ^38^, ^39^, ^40^, ^41^, ^42^ three earlier versions of studies,^43^, ^44^, ^45^ one bladder preservation study,^46^ and one small sample size study^47^). A detailed list of these excluded studies with specific justifications is provided in Table S2. We ultimately identified 20 articles describing 17 unique clinical trials,^8^, ^18^, ^19^, ^20^, ^21^, ^22^, ^23^, ^24^, ^25^, ^26^, ^27^, ^28^, ^29^, ^30^, ^31^, ^32^, ^33^ with 3 articles^34^, ^35^, ^36^ from earlier periods included for their detailed safety data and subgroup analyses of the original cohorts. These studies comprised 1977 patients.

Among the 17 unique trials, 3 were randomized controlled trials,^8^, ^26^, ^32^ and 14 were prospective single‐arm studies.^18^, ^19^, ^20^, ^21^, ^22^, ^23^, ^24^, ^25^, ^27^, ^28^, ^29^, ^30^, ^31^, ^33^ The treatment approaches included the following: one study of enfortumab vedotin (EV) as neoadjuvant therapy,^33^ two studies of MTA monotherapy,^21^, ^30^ three studies of MTA plus chemotherapy,^24^, ^26^, ^29^ three studies of ICI monotherapy,^18^, ^19^, ^31^ and eight studies of ICI plus chemotherapy.^8^, ^20^, ^22^, ^23^, ^25^, ^27^, ^28^, ^32^ Additional therapeutic strategies included one study of dual ICI therapy (PD‐L1 plus Cytotoxic T‐Lymphocyte Antigen 4 [CTLA4] inhibition)^32^ and one study combining programmed cell death protein 1 (PD‐1) inhibition with radiation therapy.^31^ The primary outcomes, safety profiles and key characteristics of each study are presented in Table 1, with supplementary details (including sex distribution, cisplatin eligibility, number of neoadjuvant therapy cycles, comprehensive adverse event profiles and funding sources) provided in Table S3. Of the included studies, Eastern Cooperative Oncology Group Performance Status (ECOG‐PS) eligibility criteria were also documented in Table S3, with 12 studies enrolling patients with ECOG‐PS ≤ 1,^8^, ^18^, ^20^, ^21^, ^22^, ^23^, ^24^, ^25^, ^26^, ^27^, ^28^, ^29^ 3 studies including those with ECOG‐PS ≤ 2^19^, ^30^, ^33^ and 2 studies having unclear documentation of performance status criteria.^31^, ^32^As shown in Table S3, the majority of studies included in our analysis (13 studies) administered four cycles of neoadjuvant chemotherapy. The pivotal NIAGARA trial^8^ also utilized four cycles of gemcitabine‐cisplatin, either with or without durvalumab, establishing this as the current reference standard for treatment duration.

**TABLE 1:** Characteristics and outcomes of clinical trials evaluating novel neoadjuvant therapies for MIBC.

Study name, reference number	Author	Year of publication	Study design^a^	Treatment arm details (no. of patients)	Control arm details (no. of patients)	Median follow‐up duration, months	Complete response (ypT0), no. (%)	Downstaging (≤ypT1), no. (%)	Radical cystectomy performed, no. (%)	R0 resection achieved, no. (%)	Overall survival, median, months	Disease‐free survival, median, months	Grade ≥3 adverse events, no. (%)	Treatment‐related deaths, no. (%)
NIAGARA (NCT03732677)^8^	Powles	2024	Phase3, RCT	Durvalumab + GC (533)	GC (530)	42.3	199 (37.3)/146 (27.5)	265 (49.7)/215 (40.6)	469 (88.0)/441 (83.2)	NA/NA	NR/NR	NR/46.1	215 (40.6) /215 (40.9)^b^	3 (0.6)/3 (0.6)
ABACUS (NCT02662309)^18^	Szabados	2022	Phase2, prospective single‐arm study	Atezolizumab (95)	NA	24.9	27 (28.4)	34 (35.8)	87 (91.6)	80 (84.2)	NR	NA	15 (15.8)	1 (1.1)
PURE‐01 (NCT02736266)^19^	Basile	2022	Phase2, prospective single‐arm study	Pembrolizumab (155)	NA	39	57 (36.8)	83 (53.5)	143 (92.3)	NA	NR	NR	7 (4.5)	0
ONO‐4538‐X41 (KCT0003804)^20^	Kim	2023	Phase2, prospective single‐arm study	Nivolumab + GC (51)	NA	24	12 (23.5)	22 (43.1)	34 (66.7)	34 (66.7)	NR	NR	NA	0
NCT00706641^21^	Hahn	2016	Phase2, prospective single‐arm study	Dasatinib (25)	NA	29	0 (0.0)	5 (20.0)	22 (88.0)	NA	NA	NA	8 (33.3)^c^	NA
LCCC 1520 (NCT02690558)^22^	Rose	2021	Phase2, prospective single‐arm study	Pembrolizumab + GC (39)	NA	15.7	14 (35.9)	22 (56.4)	38 (97.4)	34 (87.2)	NR	NR	29 (74.4)	0
NCT02989584^23^	Funt	2022	Phase2, prospective single‐arm study	Atezolizumab + GC (44)	NA	23.6	16 (36.4)	27 (61.4)	36 (81.8)	36 (81.8)	NR	NA	26 (59.1)	NA
NCT01827618^24^ ^,^ ^d^	Makrakis	2022	Phase1/2, prospective single‐arm study	Rapamycin + GC (15)	NA	46	2 (13.3)	6 (40.0)	13 (86.7)	12 (80.0)	NA	NA	NA	NA
ChiCTR2000032359^25^	Han	2023	Phase2, prospective single‐arm study	Camrelizumab + GC (40)	NA	11	13 (32.5)	16 (40.0)	30 (75.0)	30 (75.0)	NR	NR	15 (37.5)	0
NEOBLADE (ISRCTN 56349930)^26^	Hussain	2022	Phase2, RCT	Nintedanib + GC (57)	GC (63)	33.5	21 (36.8)/20 (31.7)	NA/NA	26 (45.6)/36 (57.1)	NA/NA	NR/50.6	NR/NR	53 (93.0)/50 (79.4)	0/1 (1.6)
ChiCTR2000037670^27^	Li	2024	Phase2, prospective single‐arm study	Tislelizumab + GC (65)	NA	17.1	29 (44.6)	43 (66.2)	57 (87.7)	56 (86.2)	NR	NR	39 (60.0)	1 (1.5)
NURE‐Combo (NCT04876313)^28^	Mercinelli	2024	Phase2, prospective single‐arm study	Nivolumab + Nab‐paclitaxel (31)	NA	12	10 (32.3)	22 (71.0)	28 (90.3)	26 (83.9)	NA	NA	8 (25.8)	0
NCT01222676^29^	Necchi	2018	Phase2, prospective single‐arm study	Sorafenib + GC (46)	NA	35	20 (43.5)	25 (54.3)	43 (93.5)	40 (87.0)	NR	NR	33 (71.7)	0
LCCC 0521 (NCT00380029)^30^	Pruthi	2009	Phase2, prospective single‐arm study	Erlotinib (20)	NA	24.8	5 (25.0)	7 (35.0)	20 (100)	20 (100)	NA	NA	6 (30.0)	0
RACE IT (NCT03529890)^31^	Schmid	2022	Phase2, prospective single‐arm study	Nivolumab + radiation (33)	NA	NA	12 (36.4)	18 (54.5)	27 (81.8)	NA	NA	NA	NA	NA
NEMIO (NCT03549715)^32^	Thibault	2023	Phase1/2, RCT	Tremelimumab + durvalumab + ddMVAC (58)	Durvalumab + ddMVAC (55)	6.6	27 (46.6)/27 (49.1)	35 (60.3)/39 (70.9)	58 (100)/55 (100)	NA/NA	NA/NA	NA/NA	NA/NA	NA/NA
EV‐103 (NCT03288545)^33^	PeterH	2024	Phase1b/2, prospective single‐arm study	Enfortumab vedotin (22)	NA	NA	8 (36.4)	11 (50.0)	22 (100)	NA	NR	NR	4 (18.2)	0

Abbreviations: ddMVAC, dose‐dense methotrexate, vinblastine, doxorubicin, and cisplatin; GC, gemcitabine plus cisplatin; MIBC, muscle invasive bladder cancer; NA, not available; NR, not reached; R0 = negative surgical margin; RCT, randomized controlled trial.

^a^ In the RCT, parameters for both groups are listed in the intervention/control group order with shaded delimiters.

^b^ The number of evaluable populations for complications was 530 for the intervention group and 526 for the control group.

^c^ The number of evaluable populations for complications was 24.

^d^ This clinical trial number was at phase 0/1, and no names were given in the literature for Phase 1/2.

### 3.2 Quality assessment

For the three randomized controlled trials, the RoB2 assessment demonstrated a low risk of bias across most domains. The ROBINS‐I assessment of non‐randomized studies revealed predominantly a low risk of bias across most domains, with moderate risk noted in confounding (Domain 1) for several studies.^20^, ^21^, ^22^, ^23^, ^24^, ^25^, ^28^, ^30^, ^31^, ^33^ This moderate risk was primarily attributed to insufficient documentation of the patient selection process, including unclear criteria for cisplatin eligibility/ineligibility and inadequate explanation of reasons for chemotherapy ineligibility or refusal in several studies.

Publication bias was evaluated using a Doi plot for pCR rates. The LFK index of −1.872 indicated minor asymmetry, suggesting potential small‐study effects but no substantial publication bias that would significantly impact our findings. Detailed quality assessments and publication bias analysis are presented in Figures S2 and S3, respectively.

### 3.3 Meta‐analysis results

**FIGURE 1:**
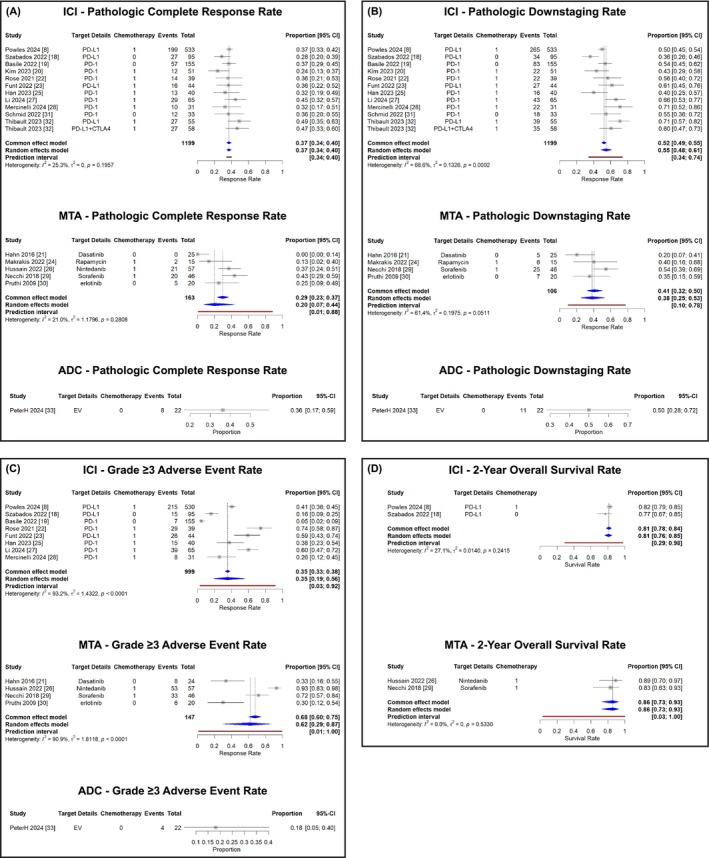
Meta‐analysis of neoadjuvant therapy outcomes in muscle‐invasive bladder cancer (A: pathologic complete response rates, B: pathologic downstaging rates, C: grade ≥3 adverse event rates, and D: 2‐year overall survival rates).

Initial analysis was associated with pathologic complete response rates of 37% (95% CI, 34%–40%) for ICI‐based treatments (*I* ^2^ = 25%, *P* = 0.20), 20% (95% CI, 7%–44%) for MTA‐based treatments (*I* ^2^ = 21%, *P* = 0.28) and 36% (95% CI, 19%–58%) for EV therapy (Figure 1A). Pathologic downstaging was observed in 55% (95% CI, 48%–61%) of patients receiving ICI‐based therapy (*I* ^2^ = 69%, *P* = 0.0002), 38% (95% CI, 25%–53%) of those receiving MTA‐based therapy (*I* ^2^ = 61%, *P* = 0.051) and 50% (95% CI, 28%–72%) of those receiving EV therapy (Figure 1B). To address the substantial heterogeneity observed, particularly in ICI downstaging rates (*I* ^2^ = 69%, *P* = 0.0002), we examined key eligibility criteria across studies. As detailed in Table S3, important variations existed in baseline patient characteristics. Regarding performance status, 12 studies enrolled patients with ECOG‐PS ≤ 1, 3 studies included those with ECOG‐PS ≤ 2 and 2 studies had unclear documentation. Cisplatin eligibility also varied considerably, with six studies (35.3%) enrolling exclusively cisplatin‐eligible patients, seven studies (41.2%) focusing on cisplatin‐ineligible or refusing populations and four studies (23.5%) not clearly reporting eligibility criteria. This heterogeneity in patient selection likely contributed to the observed variability in treatment outcomes.

The incidence of grade ≥3 adverse events was 35% (95% CI, 19%–56%) with ICI‐based treatments (*I* ^2^ = 93%, *P* < 0.001), 62% (95% CI, 29%–87%) with MTA‐based treatments (*I* ^2^ = 91%, *P* < 0.001) and 18% (95% CI, 5%–40%) with EV therapy (Figure 1C). Two‐year overall survival rates were 81% (95% CI, 76%–85%) for ICI‐based regimens (*I* ^2^ = 27%, *P* = 0.24) and 86% (95% CI, 73%–93%) for MTA‐based regimens (*I* ^2^ = 0%, *P* = 0.53) (Figure 1D).

Forest plot analyses of secondary outcomes including 2‐year disease‐free survival, cystectomy rate and R0 resection rate for each regimen are presented in Figures S4, S5 and S6. Forest plots demonstrating the incidence of grade ≥3 common adverse events (anaemia, constipation, diarrhoea, fatigue, nausea, neutropenia and thrombocytopenia) stratified by treatment type are shown in Figure S7.

Regarding surgical feasibility, across the included studies, the proportion of patients initially planned for cystectomy who ultimately underwent the procedure varied considerably. As shown in Table 1, cystectomy rates ranged from 45.6% to 100%, with a median rate of 88.0%. In the NIAGARA trial,^8^ 88.0% of patients in the durvalumab plus chemotherapy arm proceeded to cystectomy. Other notable completion rates included ABACUS^18^ (91.6%) and PURE‐01^19^ (92.3%). In a sensitivity analysis including only patients who underwent radical cystectomy (Figure S8), the pT0 rate was 42% (95% CI, 39%–45%), and the downstaging rate was 62% (95% CI, 56%–69%) in the ICI group. In the MTA group, the pT0 rate was 25% (95% CI, 6%–65%), and the downstaging rate was 41% (95% CI, 27%–57%) among patients who completed radical cystectomy. For both outcomes, substantial heterogeneity was observed in the MTA group (*I* ^2^ = 77%, *P* = 0.002 for pT0; *I* ^2^ = 61%, *P* = 0.05 for downstaging), while minimal heterogeneity was noted in the ICI group for the pT0 outcome (*I* ^2^ = 0%, *P* = 0.57).

#### 3.3.1 Treatment without chemotherapy

**FIGURE 2:**
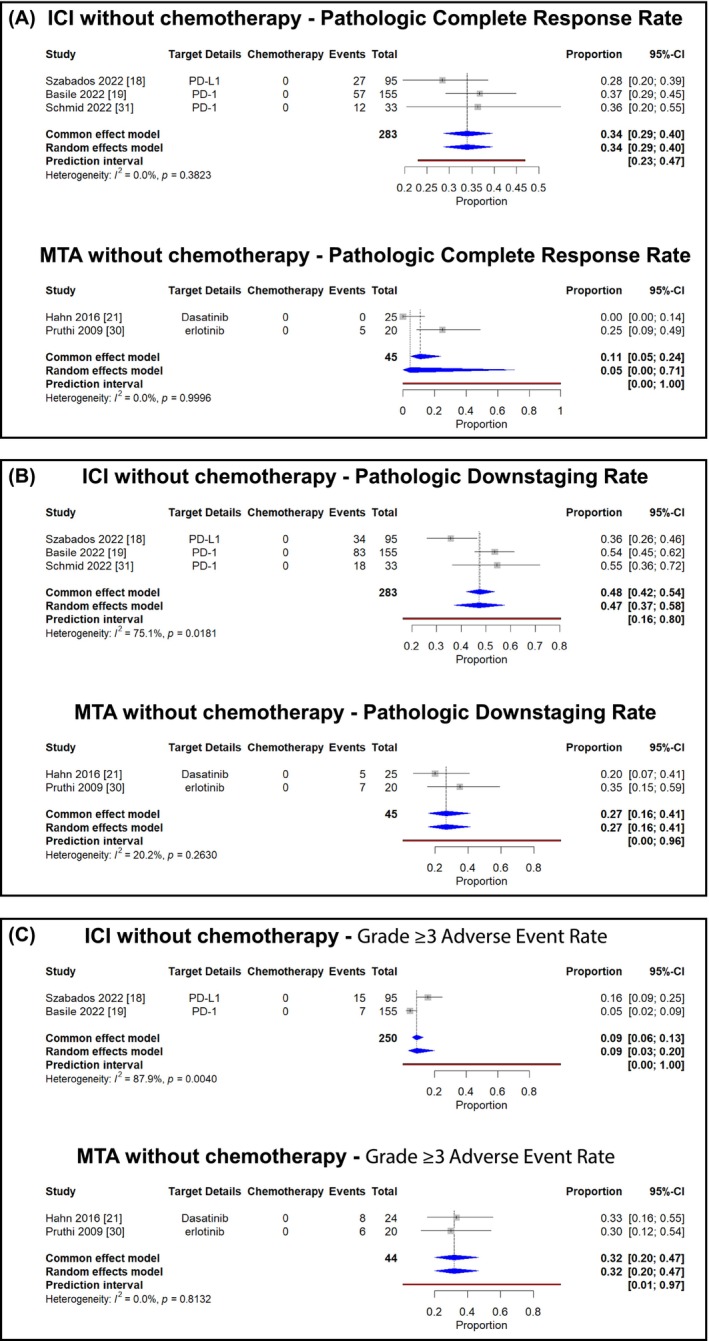
Meta‐analysis of neoadjuvant therapy outcomes with monotherapy regimens of novel agents in muscle‐invasive bladder cancer (A: pathologic complete response rates, B: pathologic downstaging rates, and C: grade ≥3 adverse event rates).

In studies evaluating monotherapy regimens, ICI monotherapy was associated with a pathologic complete response rate of 34% (95% CI, 29%–40%) in the random effects model. MTA monotherapy demonstrated a pathologic complete response rate of 5% (95% CI, 0%–71%) (Figure 2A). Pathologic downstaging rates were 47% (95% CI, 37%–58%; *I* ^2^ = 75%, *P* = 0.018) with ICI monotherapy and 27% (95% CI, 16%–41%) with MTA monotherapy (Figure 2B). Grade ≥3 adverse events occurred in 9% (95% CI, 3%–20%) of patients receiving ICI monotherapy and 32% (95% CI, 20%–47%) of patients receiving MTA monotherapy (Figure 2C). Substantial heterogeneity was noted in the analysis of pathologic downstaging rates for ICI monotherapy.

#### 3.3.2 Treatment with chemotherapy

**FIGURE 3:**
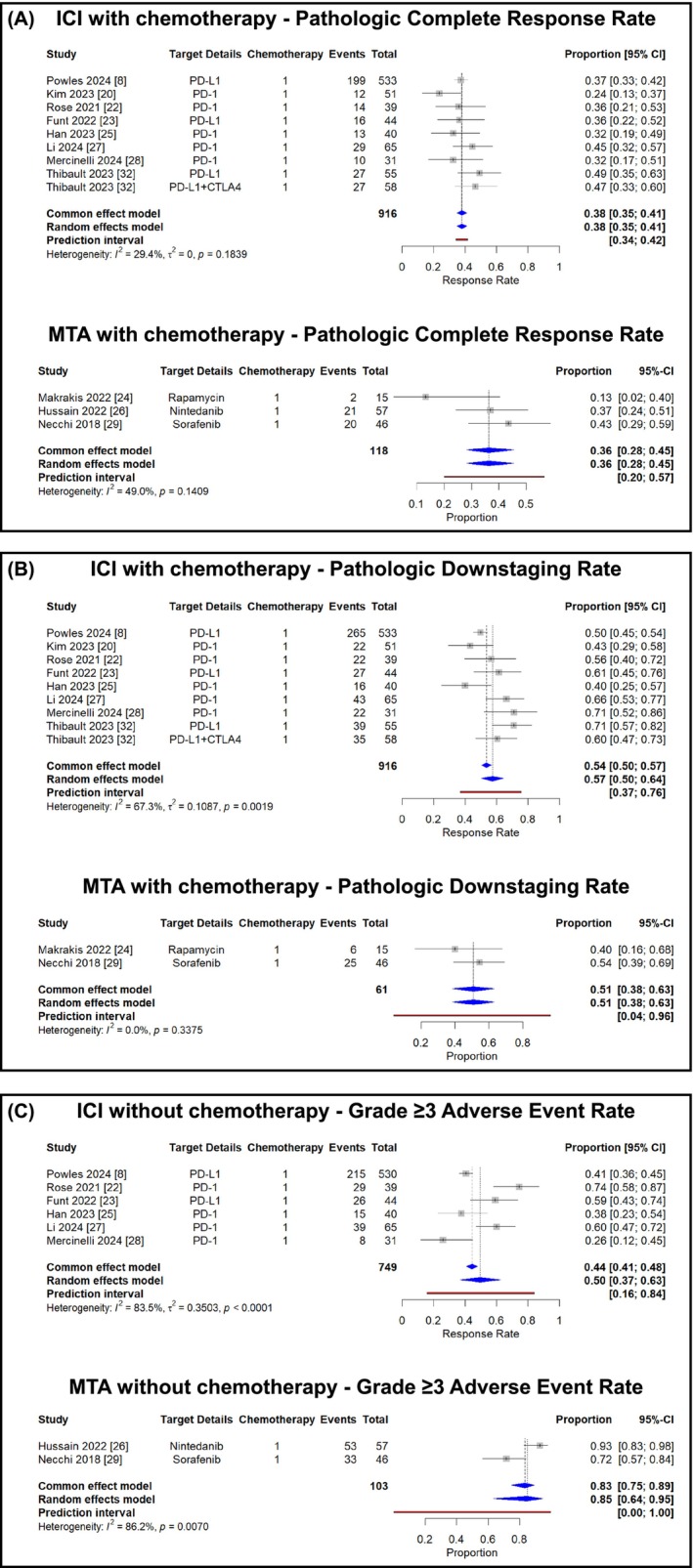
Meta‐analysis of neoadjuvant therapy outcomes with combination regimens of novel agents and chemotherapy in muscle‐invasive bladder cancer (A: pathologic complete response rates, B: pathologic downstaging rates, and C: grade ≥3 adverse event rates).

In studies evaluating combination regimens, ICI plus chemotherapy was associated with a pathologic complete response rate of 38% (95% CI, 35%–41%) in the random effects model, while MTA plus chemotherapy demonstrated a rate of 36% (95% CI, 28%–45%; *I* ^2^ = 49%, *P* = 0.14). Pathologic downstaging was observed in 57% (95% CI, 50%–64%; *I* ^2^ = 67%, *P* = 0.0019) with ICI plus chemotherapy and 51% (95% CI, 38%–63%; *I* ^2^ = 0%) with MTA plus chemotherapy. The incidence of grade ≥3 adverse events was 50% (95% CI, 37%–63%; *I* ^2^ = 84%, *P* < 0.001) with ICI plus chemotherapy and 85% (95% CI, 64%–95%; *I* ^2^ = 86%, *P* = 0.007) with MTA plus chemotherapy (Figure 3).

### 3.4 Biomarker response analysis

**FIGURE 4:**
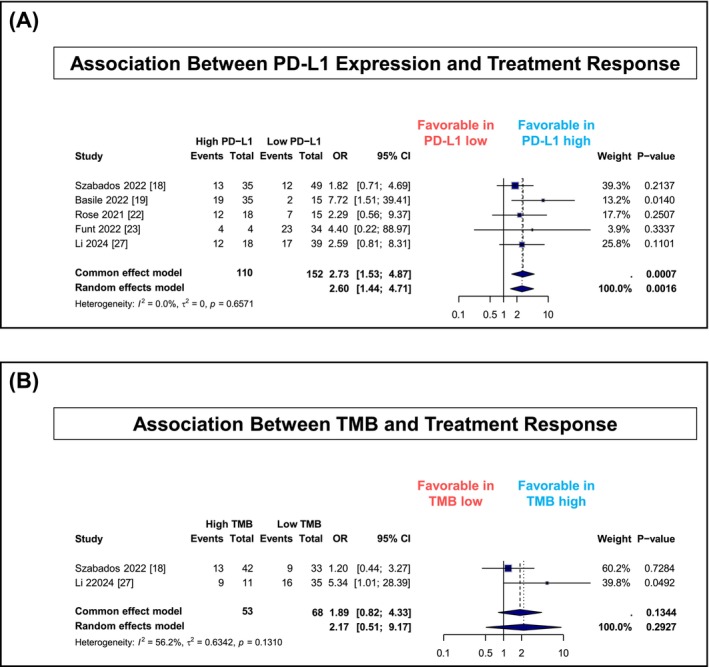
Biomarker analysis of neoadjuvant ICI therapy in muscle‐invasive bladder cancer: association of PD‐L1 expression and tumour mutational burden with pathologic response (A: PD‐L1 expression, B: TMB). ICI, immune checkpoint inhibitor; PD‐L1, programmed death‐ligand 1; TMB, tumour mutational burden.

In the analysis of PD‐L1 expression, high PD‐L1 status was significantly associated with improved pathologic complete response in the random effects model (OR, 2.60; 95% CI, 1.44–4.71; *P* = 0.0016; *I* ^2^ = 0%) (Figure 4A). Five studies contributed to this analysis, utilizing various antibody clones and positivity thresholds: SP142 (cut‐off 5%) in two studies,^18^, ^23^ 22C3 (cut‐off 10%) in two studies,^19^, ^22^ 22C3 (cut‐off 1%) in one study^20^ and SP263 (cut‐off 25%) in one study.^27^ Despite these methodological differences in PD‐L1 assessment, statistical heterogeneity was not observed (*I* ^2^ = 0%, *P* = 0.657). Individual study odds ratios ranged from 1.82 to 7.72, with the strongest association observed in the PURE‐01 trial (OR, 7.72; 95% CI, 1.51–39.41; *P* = 0.014).^19^ The association between TMB and treatment response was evaluated in two studies with 121 patients, with a nonsignificant trend suggesting potential benefit in high TMB status (OR, 2.17; 95% CI, 0.51–9.17; *P* = 0.29; *I* ^2^ = 56%) (Figure 4B). Additionally, the PURE‐1 study^19^ reported a significant non‐linear association between TMB and pathologic response to pembrolizumab (*P* = 0.022), although the detailed data were not available for inclusion in the meta‐analysis.

## 4 DISCUSSION

This systematic review and meta‐analysis examined outcomes of novel neoadjuvant therapies in MIBC, analysing data from 17 clinical trials encompassing 1977 patients. The findings suggest that novel therapeutic approaches, particularly ICI‐based regimens, were associated with favourable pathologic response rates. The NIAGARA trial^8^ contributed 1063 of the 1977 patients (53.8%) in our analysis, representing the largest and most recent randomized phase III trial in this space. This landmark study demonstrated that adding durvalumab to gemcitabine‐cisplatin improved pathologic complete response rates (37.3% vs. 27.5%) and pathologic downstaging (49.7% vs. 40.6%), establishing a new standard of care for neoadjuvant therapy in MIBC. The trial's size and rigor provide robust evidence that ICI‐chemotherapy combinations significantly outperform conventional chemotherapy alone.

ICI monotherapy was associated with pCR rates of 34% and downstaging rates of 47% (Figure 2). These outcomes appear numerically higher than the 28.6% pCR rate with conventional chemotherapy reported in a previous meta‐analysis by Petrelli et al.^2^ However, notable methodological differences exist: the prior analysis primarily included patients undergoing cystectomy, while our ITT analysis included all enrolees. In our sensitivity analysis using only patients undergoing surgery as the denominator, ICI‐based therapy achieved even more favourable downstaging (62%) and pCR rates (42%) (Figure S8), suggesting robust activity.

Comparative analysis between treatment approaches revealed potentially meaningful differences. ICI monotherapy was associated with higher response rates compared with MTA monotherapy (pCR: 34% vs. 5%; downstaging: 47% vs. 27%). When combined with chemotherapy, both approaches showed increased activity (ICI combination: pCR 38%, downstaging 57%; MTA combination: pCR 36%, downstaging 51%), suggesting potential synergy. An important observation from our analysis is the modest 4% absolute improvement in pathologic complete response rates when adding chemotherapy to ICI (34% vs. 38%). This relatively small increment deserves careful consideration, particularly given the substantially higher grade ≥3 adverse event rates with combination therapy (50% vs. 9%).

Safety profiles demonstrated marked differences across treatment approaches (Table S4). ICI‐based treatments showed a more favourable grade ≥3 adverse event rate of 35% compared with 62% for MTA‐based treatments. Analysis of toxicity patterns reveals distinct signatures: ICI monotherapy was associated with predominantly immunological adverse events and a notably low grade ≥3 event rate of 9%, while the addition of chemotherapy to ICI increased this rate substantially to 50%, primarily due to chemotherapy‐induced hematologic toxicities (neutropenia 31%–42%, thrombocytopenia 24%–34%). This pattern suggests that most severe adverse events in ICI‐chemotherapy combinations can be attributed to the chemotherapy component rather than the immunotherapy. In contrast, MTA monotherapy demonstrated target‐specific toxicity profiles (rash, fatigue) with a grade ≥3 event rate of 32%, while MTA‐chemotherapy combinations exhibited the highest toxicity burden (85%) with predominantly severe hematologic and thromboembolic complications, indicating a potentially additive toxicity pattern. EV demonstrated a unique safety profile with only 18% grade ≥3 adverse events, despite enrolling a broader patient population (ECOG‐PS ≤ 2), with fatigue (predominantly grade 1–2) and peripheral neuropathy as characteristic toxicities. These distinct safety signatures should inform treatment selection strategies as ongoing trials like EV‐303 (perioperative pembrolizumab with/without EV)^37^ and EV‐304 (EV plus pembrolizumab versus chemotherapy)^38^ further investigate optimal combinations.

Our analysis includes important context regarding the optimal number of neoadjuvant chemotherapy cycles. Patel et al.^48^ demonstrated that three and four cycles of neoadjuvant chemotherapy yielded similar pathological and survival outcomes in muscle‐invasive bladder cancer. This finding has implications for our study, particularly as ICI‐based regimens are increasingly combined with chemotherapy. The NIAGARA trial,^8^ which constitutes over half of our sample size, administered four cycles of gemcitabine‐cisplatin with or without durvalumab. Whether three cycles of ICI plus chemotherapy would yield similar outcomes to four cycles remains an important question for future research, as optimizing treatment duration could potentially reduce toxicity without compromising efficacy. While implementation of neoadjuvant therapy has improved significantly over time, Patel et al.^49^ report that barriers still exist, with some patients unable to receive optimal therapy due to renal dysfunction, clinical contraindications, or patient preference despite declining refusal rates in recent years.

This meta‐analysis is subject to several limitations. First, notable heterogeneity existed across studies regarding design and populations. As documented in Table S3, performance status eligibility varied substantially (12 studies limited to ECOG‐PS ≤ 1, 3 studies allowing ECOG‐PS ≤ 2 and 2 studies with unclear criteria). Similarly, cisplatin eligibility demonstrated significant variation, with 35.3% of studies enrolling exclusively cisplatin‐eligible patients, 41.2% focusing on cisplatin‐ineligible populations and 23.5% with unclear criteria. These variations likely contributed to the observed statistical heterogeneity and may influence clinical generalizability. Second, while our total sample of 1977 patients represents the most comprehensive analysis of novel neoadjuvant approaches in MIBC to date, important limitations must be acknowledged. The distribution of this sample is notably uneven, with the NIAGARA trial contributing 1063 patients (53.8% of the total), creating a disproportionate influence on overall estimates. This imbalance is particularly evident for emerging therapeutic approaches—antibody‐drug conjugates (*n* = 22) and dual checkpoint inhibition (*n* = 58) have limited representation, necessitating caution when interpreting findings for these specific modalities. For biomarker analyses, the sample size constraints are even more pronounced. Our TMB analysis included only 121 patients, which significantly underpowers our ability to detect modest but potentially clinically meaningful associations, explaining the observed non‐significant trend (OR, 2.17; 95% CI, 0.51–9.17). Third, while methodologically appropriate, the ITT analysis may have influenced the interpretation of response rates in trials with low surgical completion rates. For instance, in NEOBLADE, only 26 of 57 patients underwent cystectomy. Our sensitivity analysis using only patients undergoing surgery as the denominator confirmed the robustness of the primary findings but reinforces the importance of considering procedural completion rates when evaluating neoadjuvant approaches. Fourth, considerable variability was observed in biomarker assessment. PD‐L1 testing involved different antibody clones and cut‐off values, and inconsistencies were noted in outcome reporting (T0 vs. downstaging) in biomarker analyses. These discrepancies hinder direct comparisons regarding predictive biomarkers. Furthermore, assessing adverse events in combination therapy studies was limited by difficulty attributing complications specifically to novel agents versus chemotherapy, potentially affecting the accuracy of safety comparisons.

Despite these limitations, our findings have important implications for clinical practice and future research. The consistent activity observed with ICI‐based approaches, particularly in biomarker‐selected populations, suggests potential for precision medicine in neoadjuvant therapy. Analysis of biomarker‐stratified outcomes revealed that high PD‐L1 expression was significantly associated with improved pCR rates (OR, 2.60; 95% CI, 1.44–4.71), supporting PD‐L1's role as a stratification factor. However, the variability in PD‐L1 testing methods (SP142, SP263 and 22C3) and positivity thresholds (1%–25%) across studies represents a practical challenge for clinical implementation. Standardization of PD‐L1 assessment, possibly through companion diagnostics specifically validated for bladder cancer, may improve biomarker utility in patient selection. The relationship between TMB and treatment response was explored in a smaller subset (121 patients), showing a nonsignificant trend toward improved outcomes in TMB‐high patients (OR, 2.17; 95% CI, 0.51–9.17). This analysis was underpowered to detect modest but potentially meaningful associations. While limited data availability from the PURE‐01 study prevented inclusion of its detailed TMB analyses—which reported a significant non‐linear association between TMB and response to pembrolizumab^19^—our findings suggest that TMB warrants further investigation in larger, prospectively designed studies with standardized TMB assessment methodology.

## 5 CONCLUSION

This systematic review and meta‐analysis highlights the promising role of novel neoadjuvant therapies, particularly ICI‐based regimens, in MIBC. The observed high pathologic response rates, coupled with distinct safety profiles across different treatment strategies, underscore the potential for personalized treatment selection tailored to individual patient and tumour characteristics. While these findings are encouraging and suggest incorporating those novel agents into the neoadjuvant setting, further investigation in RCTs is warranted to definitively establish optimal treatment algorithms and refine patient selection based on robust predictive biomarkers.

## AUTHOR CONTRIBUTIONS

Shugo Yajima had full access to all of the data in the study and take responsibility for the integrity of the data and the accuracy of the data analysis. *Concept and design*: Shugo Yajima, Hitoshi Masuda. *Acquisition, analysis or interpretation of data*: All authors. *Drafting of the manuscript*: Shugo Yajima. *Critical review of the manuscript for important intellectual content*: All authors. *Statistical analysis:* Shugo Yajima. *Obtained funding*: N/A. *Administrative, technical or material support*: N/A. *Supervision*: Hitoshi Masuda.

## CONFLICT OF INTEREST STATEMENT

The authors have no conflicts of interest to declare.

## Supporting information


**Table S1:** PRISMA 2020 checklist.
**Figure S1:** PRISMA flow diagram of study selection.
**Table S2:** Excluded studies with detailed justifications from meta‐analysis.
**Table S3:** Baseline characteristics, treatment details, and safety data from clinical trials of novel neoadjuvant therapy in MIBC.
**Table S4:** Comparative analysis of treatment‐related Adverse Events by therapeutic class.
**Figure S2:** Risk of bias assessment of the included studies using RoB2 for randomized trials (A) and ROBINS‐I for non‐randomized studies (B).
**Figure S3:** Doi Plot analysis for pathological complete response (ypT0) rate.
**Figure S4:** Forest plot analysis of 2‐year disease‐free survival by treatment type.
**Figure S5: Forest plot analysis of cystectomy rates by treatment type**.
**Figure S6:** Forest plot analysis of R0 resection rates by treatment type.
**Figure S7:** Forest plot analysis of grade ≥3 adverse events during neoadjuvant therapy for MIBC.
**Figure S8:** Sensitivity analysis of pathologic response using radical cystectomy patients as denominator: (A) pathologic complete response rates and (B) downstaging rates.

## Data Availability

All data extracted for this systematic review and meta‐analysis came from published articles cited in our references. The complete data extraction forms, statistical analysis code and the aggregated dataset will be made available by the corresponding author upon reasonable request for academic purposes.
