# Supplementary material for: Novel neoadjuvant therapies for muscle‐invasive bladder cancer: Systematic review and meta‐analysis
Source: BJUI Compass. 2025 May 26;6(5):e70031. doi: 10.1002/bco2.70031 (PMC12105875; doi:10.1002/bco2.70031)

**Supplementary Information**

1. **Supplementary Appendix:** Search strategy for systematic review and meta-analysis
2. **eTable 1:** PRISMA 2020 checklist
3. **eFigure1:** PRISMA flow diagram of study selection
4. **eTable 2:** Excluded studies with detailed justifications from meta-analysis
5. **eTable 3:** Baseline characteristics, treatment details, and safety data from clinical trials of novel neoadjuvant therapy in MIBC
6. **eTable 4:** Comparative analysis of treatment-related Adverse Events by therapeutic class
7. **eFigure 2:** Risk of bias assessment of the included studies using RoB2 for randomized trials (A) and ROBINS-I for non-randomized studies (B)
8. **eFigure 3:** Doi Plot analysis for pathological complete response (ypT0) rate
9. **eFigure 4:** Forest plot analysis of 2-year disease-free survival by treatment type
10. **eFigure 5:** Forest plot analysis of cystectomy rates by treatment type
11. **eFigure 6:** Forest plot analysis of R0 resection rates by treatment type
12. **eFigure 7:** Forest plot analysis of grade ≥3 adverse events during neoadjuvant therapy for MIBC
13. **eFigure 8:** Sensitivity analysis of pathologic response using radical cystectomy patients as denominator: (A) pathologic complete response rates and (B) downstaging rates

**Abbreviations:** ADC = Antibody-Drug Conjugate, cN+ = node-positive clinical stage, CTLA-4 = Cytotoxic T-Lymphocyte-Associated protein 4, ECOG-PS = Eastern Cooperative Oncology Group Performance Status, EV = Enfortumab Vedotin, ICI = Immune Checkpoint Inhibitor, IQR = Interquartile Range, MIBC = Muscle Invasive Bladder Cancer, MTA = Multi-Targeted Agent, mut/Mb = mutations per megabase, NA = not available, PD-1 = Programmed cell death protein 1, PD-L1 = Programmed Death-Ligand 1, PRISMA = Preferred Reporting Items for Systematic Reviews and Meta-Analyses, RoB 2 = Risk of Bias 2, ROBINS-I = Risk Of Bias In Non-randomized Studies - of Interventions, TMB = Tumor Mutational Burden

1. **Supplementary Appendix:** Search strategy for systematic review and meta-analysis

**PubMed (MEDLINE) n=285**

(("Urinary Bladder Neoplasms"[Mesh] OR "bladder cancer*"[tiab] OR "bladder carcinoma*"[tiab] OR "bladder tumor*"[tiab] OR "bladder tumour*"[tiab] OR "bladder neoplasm*"[tiab] OR "muscle invasive bladder cancer"[tiab]) AND

("Neoadjuvant Therapy"[Mesh] OR "neoadjuvant"[tiab] OR "preoperative"[tiab] OR "presurgical"[tiab]) AND

("Antineoplastic Agents"[Mesh] OR "Drug Therapy"[Mesh] OR "Immunotherapy"[Mesh] OR "Molecular Targeted Therapy"[Mesh] OR chemotherapy[tiab] OR immunotherapy[tiab] OR "checkpoint inhibitor*"[tiab] OR "PD-1"[tiab] OR "PD-L1"[tiab] OR "CTLA-4"[tiab] OR "antibody-drug conjugate*"[tiab] OR "enfortumab vedotin"[tiab] OR "tyrosine kinase inhibitor*"[tiab] OR erdafitinib[tiab] OR "molecular targeted"[tiab] OR cisplatin[tiab] OR gemcitabine[tiab] OR MVAC[tiab] OR GC[tiab]) AND

("Cystectomy"[Mesh] OR cystectomy[tiab] OR "radical cystectomy"[tiab]) AND

("Treatment Outcome"[Mesh] OR "pathologic complete response"[tiab] OR pCR[tiab] OR ypT0[tiab] OR "survival rate"[tiab] OR "disease free survival"[tiab] OR "overall survival"[tiab] OR "progression free survival"[tiab] OR "adverse event*"[tiab] OR toxicity[tiab]) AND

("Clinical Trial"[pt] OR "Clinical Studies as Topic"[Mesh] OR "Clinical Trial" [Publication Type] OR "Randomized Controlled Trial" [Publication Type] OR trial*[tiab] OR "phase 2"[tiab] OR "phase 3"[tiab] OR "phase II"[tiab] OR "phase III"[tiab])) AND

"humans"[Mesh] NOT ("Animals"[Mesh] NOT "Humans"[Mesh]) AND

2000:2024[dp] AND English[lang]

**Cochrane Library n=213**

#1 MeSH descriptor: [Urinary Bladder Neoplasms] explode all trees

#2 ((bladder NEXT cancer) OR (bladder NEXT cancers) OR (bladder NEXT carcinoma) OR (bladder NEXT carcinomas) OR (bladder NEXT tumor) OR (bladder NEXT tumors) OR (bladder NEXT tumour) OR (bladder NEXT tumours) OR (bladder NEXT neoplasm) OR (bladder NEXT neoplasms))

#3 #1 OR #2

#4 MeSH descriptor: [Neoadjuvant Therapy] explode all trees

#5 (neoadjuvant OR preoperative OR presurgical)

#6 #4 OR #5

#7 (chemotherapy OR immunotherapy OR (checkpoint NEXT inhibitor) OR "PD-1" OR "PD-L1" OR "CTLA-4" OR (antibody NEXT drug NEXT conjugate) OR "enfortumab vedotin" OR (tyrosine NEXT kinase NEXT inhibitor) OR erdafitinib OR (molecular NEXT targeted) OR cisplatin OR gemcitabine OR MVAC OR GC)

#8 MeSH descriptor: [Cystectomy] explode all trees

#9 (cystectomy OR (radical NEXT cystectomy))

#10 #8 OR #9

#11 ("pathologic complete response" OR pCR OR ypT0 OR "survival rate" OR "disease free survival" OR "overall survival" OR "progression free survival" OR (adverse NEXT event) OR (adverse NEXT events) OR toxicity)

#12 ("Clinical Trial" OR "phase 2" OR "phase II" OR "phase 3" OR "phase III" OR randomized)

#13 #3 AND #6 AND #7 AND #10 AND #11 AND #12

with Cochrane Library publication date from Jan 2000 to Dec 2024

**Web of Science n=269**

#1 TS=("bladder cancer" OR "bladder carcinoma" OR "bladder tumor" OR "bladder tumour" OR "bladder neoplasm" OR "muscle invasive bladder cancer")

#2 TS=(neoadjuvant OR preoperative OR presurgical)

#3 TS=(chemotherapy OR immunotherapy OR "checkpoint inhibitor" OR "PD-1" OR "PD-L1" OR "CTLA-4" OR "antibody-drug conjugate" OR "enfortumab vedotin" OR "tyrosine kinase inhibitor" OR erdafitinib OR "molecular targeted" OR cisplatin OR gemcitabine OR MVAC OR GC)

#4 TS=(cystectomy OR "radical cystectomy")

#5 TS=("pathologic complete response" OR pCR OR ypT0 OR "survival rate" OR "disease free survival" OR "overall survival" OR "progression free survival" OR "adverse event*" OR toxicity)

#6 TS=(trial OR "phase 2" OR "phase II" OR "phase 3" OR "phase III" OR randomized)

#7 TS=("T2" OR "T3" OR "T4" OR "muscle invasive")

#8 (#1 AND #2 AND #3 AND #4 AND #5 AND #6 AND #7)

Refined by: DOCUMENT TYPES: ARTICLE

Timespan: 2000-2024

**Google Scholar n=58**

allintitle: ("bladder cancer" OR "bladder carcinoma") (neoadjuvant OR preoperative) ("immune checkpoint inhibitor" OR immunotherapy OR pembrolizumab OR atezolizumab OR durvalumab OR nivolumab OR "antibody drug conjugate" OR "enfortumab vedotin" OR "molecular targeted therapy") ("phase II" OR "phase III" OR "randomized" OR "randomised" OR trial) -retrospective -"case report" -review -meta-analysis -"systematic review" -"case series" -"real world" -registry -editorial -letter -comment Custom range: 2000-2024

**ClinicalTrials.gov n=114**

Condition or disease: (Bladder Cancer OR Bladder Carcinoma OR Muscle Invasive Bladder Cancer)

Other terms: ((neoadjuvant OR preoperative) AND (cT2 OR cT3 OR cT4 OR muscle-invasive))

Study Type: Interventional

Phase: Phase 2 OR Phase 3

1. **Supplementary Table 1:** PRISMA 2020 checklist

| **Section and Topic** | **Item #** | **Checklist item** | **Location where item is reported** |
| --- | --- | --- | --- |
| **TITLE** | | |  |
| Title | 1 | Identify the report as a systematic review. | Title page |
| **ABSTRACT** | | |  |
| Abstract | 2 | See the PRISMA 2020 for Abstracts checklist. | Page5, abstract |
| **INTRODUCTION** | | |  |
| Rationale | 3 | Describe the rationale for the review in the context of existing knowledge. | First to third paragraphs of Introduction |
| Objectives | 4 | Provide an explicit statement of the objective(s) or question(s) the review addresses. | Final paragraph of Introduction |
| **METHODS** | | |  |
| Eligibility criteria | 5 | Specify the inclusion and exclusion criteria for the review and how studies were grouped for the syntheses. | Methods section 2.2 Study Selection |
| Information sources | 6 | Specify all databases, registers, websites, organisations, reference lists and other sources searched or consulted to identify studies. Specify the date when each source was last searched or consulted. | Methods section 2.1 Literature Search Strategy |
| Search strategy | 7 | Present the full search strategies for all databases, registers and websites, including any filters and limits used. | Methods section 2.1, referring to complete strategy in Supplementary Appendix |
| Selection process | 8 | Specify the methods used to decide whether a study met the inclusion criteria of the review, including how many reviewers screened each record and each report retrieved, whether they worked independently, and if applicable, details of automation tools used in the process. | First sentence of Methods section 2.4 Data Extraction and Quality Assessment |
| Data collection process | 9 | Specify the methods used to collect data from reports, including how many reviewers collected data from each report, whether they worked independently, any processes for obtaining or confirming data from study investigators, and if applicable, details of automation tools used in the process. | Methods section 2.4 Data Extraction and Quality Assessment |
| Data items | 10a | List and define all outcomes for which data were sought. Specify whether all results that were compatible with each outcome domain in each study were sought (e.g. for all measures, time points, analyses), and if not, the methods used to decide which results to collect. | Methods section 2.3 Outcomes |
|  | 10b | List and define all other variables for which data were sought (e.g. participant and intervention characteristics, funding sources). Describe any assumptions made about any missing or unclear information. | Methods section 2.3 Outcomes |
| Study risk of bias assessment | 11 | Specify the methods used to assess risk of bias in the included studies, including details of the tool(s) used, how many reviewers assessed each study and whether they worked independently, and if applicable, details of automation tools used in the process. | Methods section 2.4 describing use of RoB 2 and ROBINS-I tools |
| Effect measures | 12 | Specify for each outcome the effect measure(s) (e.g. risk ratio, mean difference) used in the synthesis or presentation of results. | Methods section 2.5 Statistical Analysis |
| Synthesis methods | 13a | Describe the processes used to decide which studies were eligible for each synthesis (e.g. tabulating the study intervention characteristics and comparing against the planned groups for each synthesis (item #5)). | Methods section 2.5 Statistical Analysis |
|  | 13b | Describe any methods required to prepare the data for presentation or synthesis, such as handling of missing summary statistics, or data conversions. | Methods section 2.5 Statistical Analysis |
|  | 13c | Describe any methods used to tabulate or visually display results of individual studies and syntheses. | Methods section 2.5 Statistical Analysis |
|  | 13d | Describe any methods used to synthesize results and provide a rationale for the choice(s). If meta-analysis was performed, describe the model(s), method(s) to identify the presence and extent of statistical heterogeneity, and software package(s) used. | Methods section 2.5 Statistical Analysis |
|  | 13e | Describe any methods used to explore possible causes of heterogeneity among study results (e.g. subgroup analysis, meta-regression). | Methods section 2.5 Statistical Analysis |
|  | 13f | Describe any sensitivity analyses conducted to assess robustness of the synthesized results. | Methods section 2.5 Statistical Analysis |
| Reporting bias assessment | 14 | Describe any methods used to assess risk of bias due to missing results in a synthesis (arising from reporting biases). | Methods section 2.5 Statistical Analysis |
| Certainty assessment | 15 | Describe any methods used to assess certainty (or confidence) in the body of evidence for an outcome. | Methods section 2.5 Statistical Analysis" |
| **RESULTS** | | |  |
| Study selection | 16a | Describe the results of the search and selection process, from the number of records identified in the search to the number of studies included in the review, ideally using a flow diagram. | Results section 3.1 Study Selection |
|  | 16b | Cite studies that might appear to meet the inclusion criteria, but which were excluded, and explain why they were excluded. | Results section 3.1 Study Selection |
| Study characteristics | 17 | Cite each included study and present its characteristics. | Final paragraph of Results section 3.1 and Table 1 |
| Risk of bias in studies | 18 | Present assessments of risk of bias for each included study. | Results section 3.2 Quality Assessment |
| Results of individual studies | 19 | For all outcomes, present, for each study: (a) summary statistics for each group (where appropriate) and (b) an effect estimate and its precision (e.g. confidence/credible interval), ideally using structured tables or plots. | Table 1 |
| Results of syntheses | 20a | For each synthesis, briefly summarise the characteristics and risk of bias among contributing studies. | Results sections 3.3, 3.3.1, 3.3.2 |
|  | 20b | Present results of all statistical syntheses conducted. If meta-analysis was done, present for each the summary estimate and its precision (e.g. confidence/credible interval) and measures of statistical heterogeneity. If comparing groups, describe the direction of the effect. | Results sections 3.3, 3.3.1, 3.3.2 |
|  | 20c | Present results of all investigations of possible causes of heterogeneity among study results. | Results sections 3.3, 3.3.1, 3.3.2 |
|  | 20d | Present results of all sensitivity analyses conducted to assess the robustness of the synthesized results. | Results sections 3.3, 3.3.1, 3.3.2 |
| Reporting biases | 21 | Present assessments of risk of bias due to missing results (arising from reporting biases) for each synthesis assessed. | Second paragraph of Results section 3.2 analyzing Doi plot |
| Certainty of evidence | 22 | Present assessments of certainty (or confidence) in the body of evidence for each outcome assessed. | Results section 3.2 |
| **DISCUSSION** | | |  |
| Discussion | 23a | Provide a general interpretation of the results in the context of other evidence. | First and second paragraphs of Discussion |
|  | 23b | Discuss any limitations of the evidence included in the review. | Limitation section in Discussion |
|  | 23c | Discuss any limitations of the review processes used. | Limitation section in Discussion |
|  | 23d | Discuss implications of the results for practice, policy, and future research. | Conclusion section |
| **OTHER INFORMATION** | | |  |
| Registration and protocol | 24a | Provide registration information for the review, including register name and registration number, or state that the review was not registered. | Methods section first paragraph |
|  | 24b | Indicate where the review protocol can be accessed, or state that a protocol was not prepared. | Methods section first paragraph |
|  | 24c | Describe and explain any amendments to information provided at registration or in the protocol. | N/A |
| Support | 25 | Describe sources of financial or non-financial support for the review, and the role of the funders or sponsors in the review. | Title Page |
| Competing interests | 26 | Declare any competing interests of review authors. | Title Page |
| Availability of data, code and other materials | 27 | Report which of the following are publicly available and where they can be found: template data collection forms; data extracted from included studies; data used for all analyses; analytic code; any other materials used in the review. | Title Page |

*From:*  Page MJ, McKenzie JE, Bossuyt PM, Boutron I, Hoffmann TC, Mulrow CD, et al. The PRISMA 2020 statement: an updated guideline for reporting systematic reviews. BMJ 2021;372:n71. doi: 10.1136/bmj.n71

1. **eFigure1:** PRISMA flow diagram of study selection


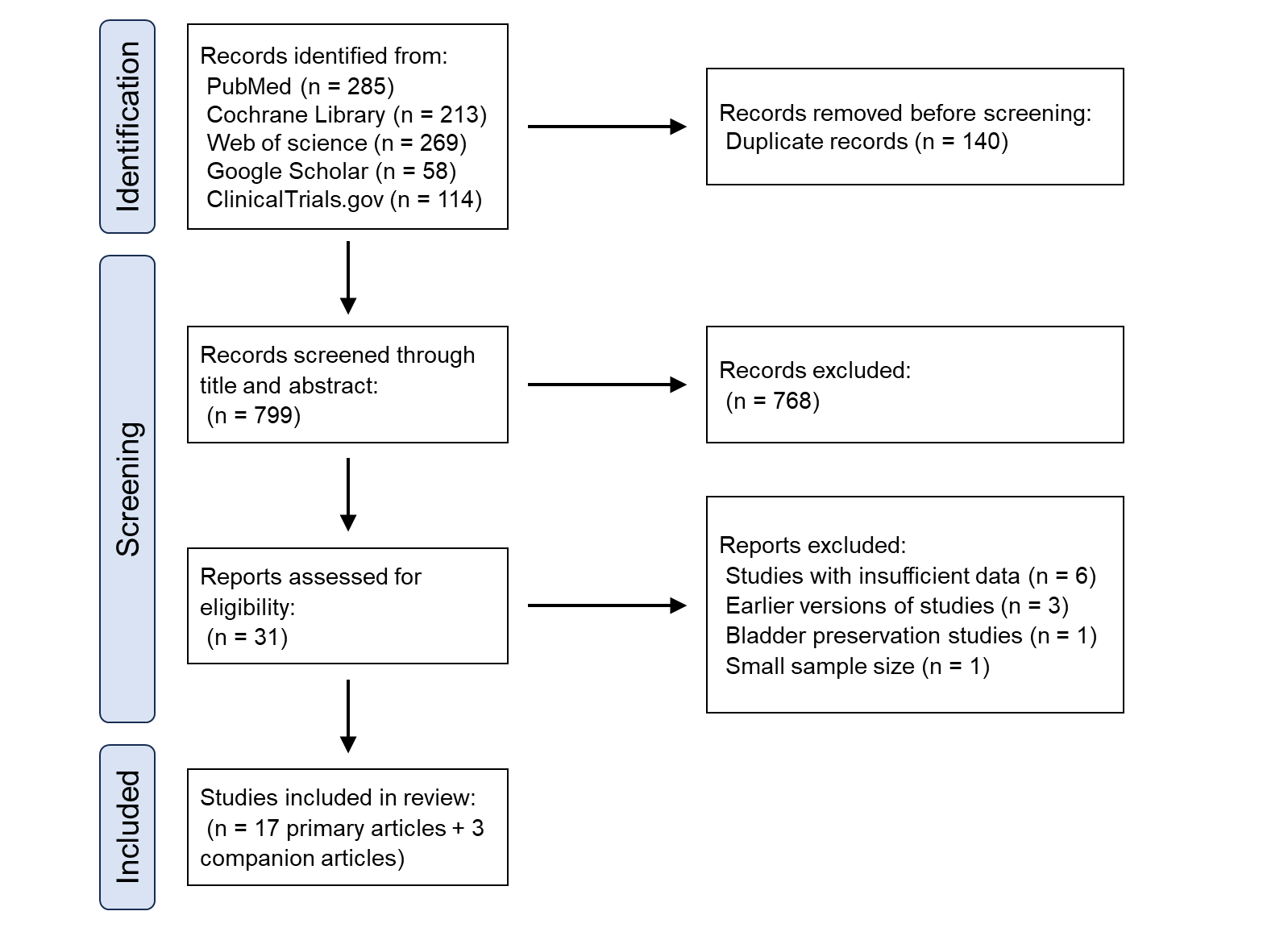


1. **eTable 2:** Excluded studies with detailed justifications from meta-analysis

| **Reference** | **Authors** | **Year** | **Title** | **Reason for Exclusion** |
| --- | --- | --- | --- | --- |
| 37 | Galsky MD, et al. | 2021 | Perioperative pembrolizumab therapy in muscle-invasive bladder cancer: Phase III KEYNOTE-866 and KEYNOTE-905/EV-303 | Insufficient data (study protocol/design only) |
| 38 | Hoimes CJ, et al. | 2023 | Perioperative enfortumab vedotin (EV) plus pembrolizumab (pembro) versus chemotherapy in cisplatin-eligible patients (pts) with muscle-invasive bladder cancer (MIBC): Phase 3 KEYNOTE-B15/EV-304 | Insufficient data (trial in progress abstract only) |
| 39 | Schmid SC, et al. | 2020 | Radiation therapy before radical cystectomy combined with immunotherapy in locally advanced bladder cancer - study protocol of a prospective, single arm, multicenter phase II trial (RACE IT) | Insufficient data (study protocol only without outcome data) |
| 40 | Thibault C, et al. | 2020 | Open-label phase II to evaluate the efficacy of NEoadjuvant dose-dense MVAC In cOmbination with durvalumab and tremelimumab in muscle-invasive urothelial carcinoma: NEMIO | Insufficient data (study protocol only without outcome data) |
| 41 | Petrausch U, et al. | 2023 | Novel sequential treatment strategy for patients with muscle-invasive bladder cancer (MIBC): intravesical recombinant BCG, followed by neoadjuvant chemoimmunotherapy, radical cystectomy plus pelvic lymphadenectomy and adjuvant immunotherapy - protocol of a multicentre, single arm phase 2 trial (SAKK 06/19) | Insufficient data (study protocol only without outcome data) |
| 42 | Necchi A, et al. | 2024 | TAR-200 plus cetrelimab (CET) or CET alone as neoadjuvant therapy in patients (pts) with muscle-invasive bladder cancer (MIBC) who are ineligible for or refuse neoadjuvant cisplatin-based chemotherapy (NAC): Interim analysis of SunRISe-4 (SR-4) | Insufficient data (preliminary interim analysis without complete pathological outcome assessment) |
| 43 | Bandini M, et al. | 2021 | Predicting the Pathologic Complete Response After Neoadjuvant Pembrolizumab in Muscle-Invasive Bladder Cancer | Earlier version of included study (PURE-01 trial with preliminary data; ref. 19 contains full dataset) |
| 44 | Necchi A, et al. | 2020 | Updated Results of PURE-01 with Preliminary Activity of Neoadjuvant Pembrolizumab in Patients with Muscle-invasive Bladder Carcinoma with Variant Histologies | Earlier version of included study (PURE-01 trial with preliminary data; ref. 19 contains full dataset) |
| 45 | Bandini M, et al. | 2020 | Does the administration of preoperative pembrolizumab lead to sustained remission post-cystectomy? First survival outcomes from the PURE-01 study | Earlier version of included study (PURE-01 trial with preliminary data; ref. 19 contains full dataset) |
| 46 | Galsky MD, et al. | 2023 | Gemcitabine and cisplatin plus nivolumab as organ-sparing treatment for muscle-invasive bladder cancer: a phase 2 trial | Bladder preservation study (no radical cystectomy as primary intervention) |
| 47 | Narayan V, et al. | 2016 | Cisplatin, Gemcitabine, and Lapatinib as Neoadjuvant Therapy for Muscle-Invasive Bladder Cancer | Small sample size study (n < 10) |

1. **eTable 3:** Baseline characteristics, treatment details, and safety data from clinical trials of novel neoadjuvant therapy in MIBC

| Study name, reference number | Age, median (range), year | Male sex, No. (%) | ECOG-PS, cutoff value for eligibility | Cisplatin Eligibility | Clinical stage II/III/IV, No. (%) | Node-positive disease (cN+), No. (%) | Protocol treatment cycles | Details of adjuvant therapy | PD-L1 Assessment | | TMB | | Notable treatment-related adverse events | Funding sources |
| --- | --- | --- | --- | --- | --- | --- | --- | --- | --- | --- | --- | --- | --- | --- |
|  |  |  |  |  |  |  |  |  | Antibody clone | Cut-off | Cut-off | Rate of TMB high (%) |  |  |
| NIAGARA (NCT03732677)^8^ | 65 (34-84) | 437 (82.0) | ≤1 | eligible | 215 (40.3)/318 (59.7)/0 | 28 (5.3) | 4 | 383/469 (81.7%) patients underwent adjuvant therapy, Durvalumab 1500mg every 4 weeks for up to 8 cycles | SP263 | 25% | NA | NA | Common complications were hypothyroidism (10.4%), hyperthyroidism (2.5%), and dermatitis/rash (2.3%) | AstraZeneca, National Institute for Health and Care Research Barts Biomedical Research Centre, Queen Mary University of London, Roche, J. Bull and M. Jacobson, Cancer Research UK, UK Experimental Cancer Medicine Network, La Roche-Hoffmann |
| ABACUS (NCT02662309)^18^ | 73 (53-87) | 81 (85.3) | ≤1 | ineligible or refused | 70 (73.7)/17 (17.9)/8 (8.4) | 0 | 2 | NA | SP142 | 5% | 10mut/Mb | 56.0 | Common complications were fatigue (20%), decreased appetite (6%), and transaminitis (6%). Grade 3-5 treatment-related adverse events included transaminitis (3%), fatigue (1%), and decreased appetite (1%). | Queen Mary University of London, Roche, J. Bull and M. Jacobson, Cancer Research UK, UK Experimental Cancer Medicine Network, La Roche-Hoffmann |
| PURE-01 (NCT02736266)^19^ | 68 (62-74) | 135 (87.1) | ≤2 | eligible | 75 (48.4)/NA/NA | NA | 3 | Nine patients received adjuvant cisplatin-based chemotherapy | 22C3 | 10% | 15mut/Mb | 20 | Grade 3-4 treatment-related adverse events occurred in 7 patients (4.5%), including transaminase elevation (leading to pembrolizumab discontinuation in one patient), hyperkalemia, and diarrhea. | Merck & Co., Inc. , Associazione Italiana per la Ricerca sul Cancro (AIRC), grant number: MFAG 2017 Id.20617 |
| ONO-4538-X41 (KCT0003804)^20^ | 66 (48-84) | 43 (84.3) | ≤1 | eligible | 33 (64.7)/13 (25.5)/5 (9.8) | 0 | 3 | Not performed | 22C3 | 1% | NA | NA | Grade 3-4 events included neutropenia (31.4%), thrombocytopenia (23.5%), anemia (5.9%), and infection (3.9%). Grade 1-2 skin rash and pruritus were the only immune-related adverse events reported. | Samsung Medical Center Research Fund (OTA1602441, OTA1702441), Ono Pharma Korea. Study drugs, nivolumab and gemcitabine were provided by Ono Pharma and Dong-A ST, respectively. |
| NCT00706641^21^ | 62 (47-83) | 20 (80.0) | ≤1 | ineligible or refused | 17 (70.8)/7 (29.2)/0 | NA | A median duration of 4.1 weeks of once-daily administration | Adjuvant chemotherapy was permitted at physician's discretion, but specific details were not reported | NA | NA | NA | NA | Grade 3-4 events included fatigue (8%), dyspnea (8%), enteric fistula (8%), pulmonary embolism (8%), while Grade 3-4 hematologic toxicities were rare with only anemia (4%). | Bristol-Myers Squibb |
| LCCC 1520 (NCT02690558)^22^ | 66 (45-82) | 32 (82.1) | ≤1 | eligible | 28 (71.8)/9 (23.1)/2 (5.1) | NA | 4 | NA | 22C3 | 10% | NA | NA | Common adverse events were hypothyroidism (13.3%) and hyperthyroidism (6.1%). Grade 3-4 hematologic toxicities included thrombocytopenia (34%), neutropenia (40%), and anemia (10%). | no funding |
| NCT02989584^23^ | 65 (58-69) | 33 (75.0) | ≤1 | eligible | 31 (79.5)/7 (17.9)/1 (2.6) | 0 | 4 | NA | SP142 | 5% | NA | NA | Grade 3-4 adverse events included neutropenia (36%), lymphopenia (16%), and anemia (11%). Grade 3 immune-related adverse events occurred in 11% of patients, with 5% requiring systemic steroids. | Genentech/Roche, NIH/NCI Cancer Center Support Grant P30 CA008748, NIH/NCI P50 CA221745 SPORE in Bladder Cancer |
| NCT01827618^24, a^ | 67 (51-86) | 12 (80.0) | ≤1 | eligible | NA | NA | 4 | NA | NA | NA | NA | NA | Grade 3-4 adverse events included thrombocytopenia (38%), neutropenia (24%), and anemia (19%) in the combined phase I-II cohorts. | no funding |
| ChiCTR2000032359^25^ | 67.5 (50-81) | 23 (57.5) | ≤1 | eligible | 22 (55.0)/15 (37.5)/3 (7.5) | 5 (12.5) | 3 | NA | 22C3 | NA | NA | NA | Grade 3-4 adverse events included neutropenia (25.6%), leukopenia (16.2%), and anemia (7.0%). Immune-related adverse events were all grade 1-2, with rash (16.3%) and hypothyroidism (11.6%). | The Capital Health Research and Development of Special Funding, Grant/Award Number: 2022-1-4021 |
| NEOBLADE (ISRCTN 56349930)^26^ | 67 (62-75) | 42 (73.7) | ≤1 | eligible | 35 (61.4)/17 (29.8)/5 (8.8) | NA | 4 | NA | NA | NA | NA | NA | Most common grade 3 or worse events were thromboembolic events (30%), decreased neutrophil count (39%), and hypertension (16%) | no funding |
| ChiCTR2000037670^27^ | 64 (58-68) | 57 (87.7) | ≤1 | eligible | 38/21/6 | 0 | 4 | Not performed | SP263 | 25% | 14.8mut/Mb | 23.9 | Common adverse events were hematologic toxicities. Grade 3-4 events included neutropenia (41.5%), thrombocytopenia (33.8%), and anemia (15.4%). | National Key Research and Development Program of China, National Natural Science Foundation of China, Sun Yat-Sen Memorial Hospital Clinical Research 5010 Program, Natural Science Foundation of Guangdong, Sun Yat-sen University, Guangdong Provincial Clinical Research Center for Urological Diseases, BeiGene |
| NURE-Combo (NCT04876313)^28^ | 64.7 (60-69.1) | 23 (74.2) | ≤1 | ineligible or refused | 19 (61.3)/7 (22.6)/5 (16.1) | 2 (6.5) | 4 | 22 patients received adjuvant nivolumab, with a total of 13 planned cycles | NA | NA | NA | NA | Common complications included gastrointestinal disorders (45.2%), asthenia (38.7%), and paresthesia (29%). Grade 3 treatment-related adverse events occurred in 8 patients (25.8%), primarily involving neutropenia, transaminase increase, and acute renal injury. | Bristol Myers Squibb, Associazione Italiana per la Ricerca sul Cancro (AIRC) |
| NCT01222676^29^ | 62.5 (57-67) | 39 (84.8) | ≤1 | eligible | 28 (60.9)/16 (34.8)/2 (4.3) | NA | 4 | Not performed | NA | NA | NA | NA | Hematologic toxicities were predominant, with grade 3-4 thrombocytopenia in 28.3% and neutropenia in 21.7% of patients. | Bayer AG, Fondazione IRCCS Istituto Nazionale dei Tumori |
| LCCC 0521 (NCT00380029)^30^ | 67.1 (NA) | 15 (75.0) | ≤2 | Not reported | NA | NA | 4 | Twelve patients continued erlotinib in the adjuvant phase with a mean duration of 29 weeks (range, 5-84 weeks). | NA | NA | NA | NA | Most common side effect was rash in 15 patients (75%), with 4 patients experiencing grade 3 severity. Fatigue and anorexia were also reported in 6 patients each. | OSI Pharmaceuticals |
| RACE IT (NCT03529890)^31^ | NA | NA | NA | ineligible or refused | NA | 10 (30.3) | 4 | NA | NA | NA | NA | NA | Treatment-related adverse events occurred in 54.5% of patients, mostly grade 1-2. Common events included thyroid disorders (15.2%), gastrointestinal disorders (15.2%), and skin reactions (33.3%). Treatment discontinuation due to adverse events occurred in 25.8% of patients. | Bristol Myers Squibb |
| NEMIO (NCT03549715)^32^ | 64 (IQR: 58-70) | NA | NA | eligible | NA | NA | 4 | NA | NA | NA | NA | NA | All patients experienced at least one treatment-related adverse event, with 41% experiencing grade 3 events. The most common grade 3 adverse events were neutropenia (12.6%), anemia (9.2%), and acute kidney injury (6.7%). | AstraZeneca |
| EV-103 (NCT03288545)^33^ | 74.5 (NA) | NA | ≤2 | ineligible or refused | 15 (68.2)/6 (27.3)/1 (4.5) | NA | 3 | 36.4% of patients received subsequent cancer-related therapy | NA | NA | NA | NA | The most common treatment-related adverse events were fatigue (45.5%), dysgeusia (36.4%), and alopecia (31.8%). Grade ≥3 treatment-related adverse events occurred in 18.2% of patients. | Seagen Inc. (acquired by Pfizer in Dec. 2023), Astellas Pharma |

ECOG-PS = Eastern Cooperative Oncology Group Performance Status, IQR = interquartile range, MIBC = muscle invasive bladder cancer, mut/Mb = mutations per megabase, NA = not available, cN+ = node-positive clinical stage, PD-L1 = Programmed Death-Ligand 1, TMB = Tumor Mutational Burden
Annotation: a: This clinical trial number was at phase 0/1, and no names were given in the literature for Phase 1/2.

1. **eTable4:** Comparative analysis of treatment-related Adverse Events by therapeutic class

| **Treatment Type** | **Grade ≥3 AE Rate**  **(95% CI)** | **Most Common Grade ≥3 Toxicities** | **Characteristic Toxicity Pattern** | **Agent-Specific Notable Toxicities** |
| --- | --- | --- | --- | --- |
| **ICI Monotherapy** | 9% (3%-20%) | • Transaminitis  • Diarrhea  • Hyperkalemia | **Immune-mediated events**:  • Predominantly low-grade thyroid dysfunction  • Skin reactions  • Generally mild-moderate severity | • **Pembrolizumab** (PURE-01)^19,36^: Transaminase elevation leading to discontinuation  • **Atezolizumab** (ABACUS)^18,34^: Transaminitis (3%), fatigue (1%) |
| **ICI + Chemotherapy** | 50% (37%-63%) | • Neutropenia (31-42%)  • Thrombocytopenia (24-34%)  • Anemia (6-15%) | **Mixed pattern**:  • Chemotherapy-related hematologic toxicities  • ICI-related immune events (generally less severe) | • **Durvalumab+GC** (NIAGARA)^8^: Hypothyroidism (10.4%), hyperthyroidism (2.5%), rash (2.3%)  • **Nivolumab+GC** (ONO-4538)^20^: Neutropenia (31.4%), thrombocytopenia (23.5%)  • **Tislelizumab+GC**^27^: Neutropenia (41.5%), thrombocytopenia (33.8%) |
| **MTA Monotherapy** | 32% (20%-47%) | • Fatigue (8%)  • Rash  • Diarrhea | **Target-specific events**:  • Drug-specific toxicity profiles  • Generally less hematologic toxicity than combinations | • **Dasatinib**^21^: Fatigue (8%), dyspnea (8%), enteric fistula (8%)  • **Erlotinib**^30^: Rash (75%, grade 3 in 20%), fatigue, anorexia |
| **MTA + Chemotherapy** | 85% (64%-95%) | • Thrombocytopenia (28-38%)  • Neutropenia (22-39%)  • Anemia (19%)  • Thromboembolic events (30%) | **High severity pattern**:  • Additive toxicity profile  • Predominantly severe hematologic events | • **Sorafenib+GC**^29^: Thrombocytopenia (28.3%), neutropenia (21.7%)  • **Nintedanib+GC**^26^: Thromboembolic events (30%), neutropenia (39%)  • **Rapamycin+GC**^24^: Thrombocytopenia (38%), neutropenia (24%) |
| **ADC**  (Enfortumab Vedotin) | 18% (5%-40%) | • Fatigue  • Peripheral neuropathy | **Unique profile**:  • Lower grade ≥3 AE rate despite broader patient eligibility (ECOG ≤2) | • **Enfortumab Vedotin**^33^: Fatigue (45.5%, mostly grade 1-2), dysgeusia (36.4%), alopecia (31.8%) |

ADC = Antibody-Drug Conjugate; AE = Adverse Event; CI = Confidence Interval; ECOG-PS = Eastern Cooperative Oncology Group Performance Status; GC = Gemcitabine plus Cisplatin; ICI = Immune Checkpoint Inhibitor; MIBC = Muscle-Invasive Bladder Cancer; MTA = Molecular Targeted Agent

**Note**: The percentages represent the incidence rates reported across the included studies. Grade ≥3 AE rates for each treatment category are from the meta-analysis results.

1. **eFigure 2:** Risk of bias assessment of the included studies using RoB2 for randomized trials (A) and ROBINS-I for non-randomized studies (B)
2. RoB2 assessment


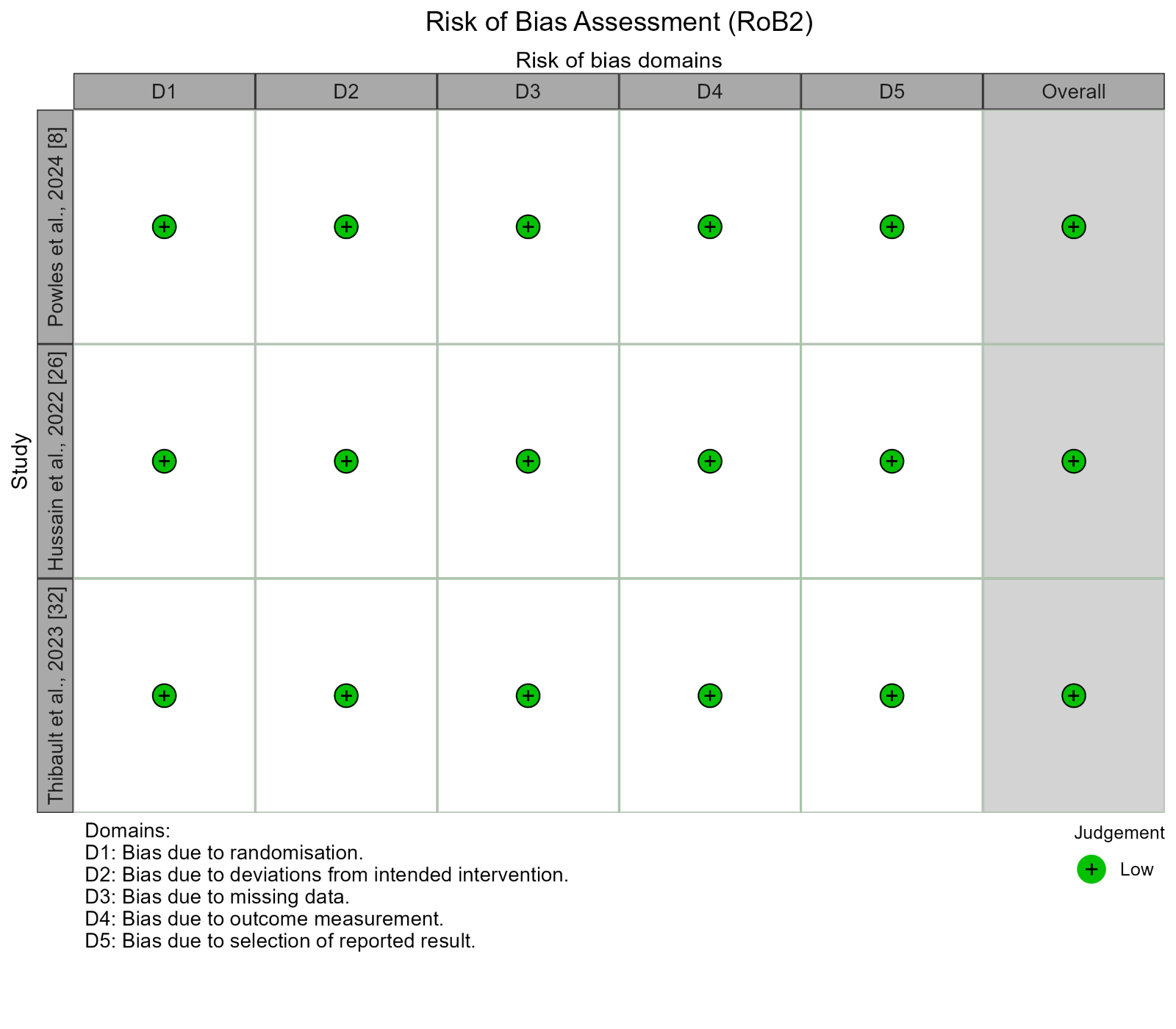


1. ROBINS-I assessment


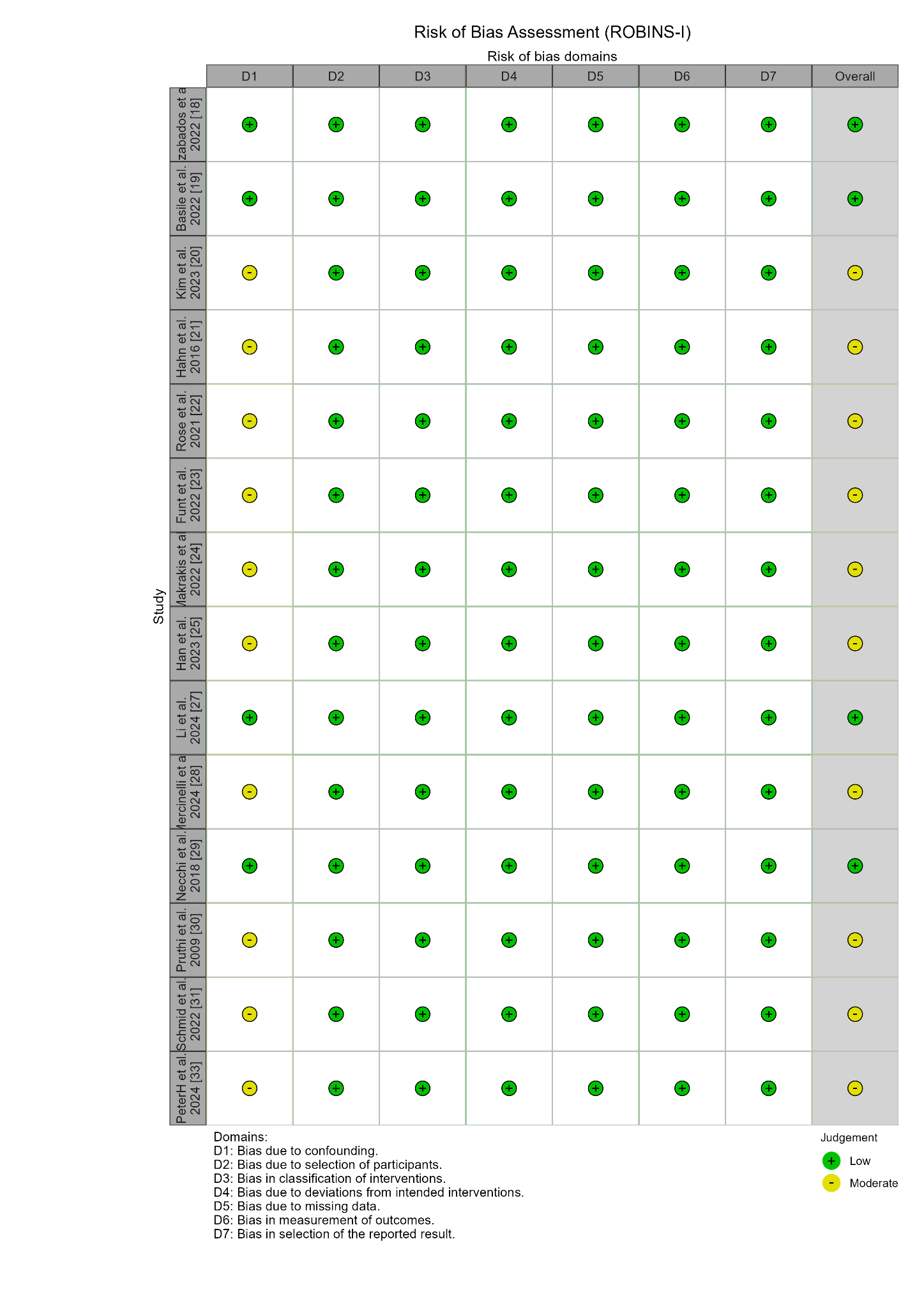


1. **eFigure 3:** Doi Plot analysis for pathological complete response (ypT0) rate


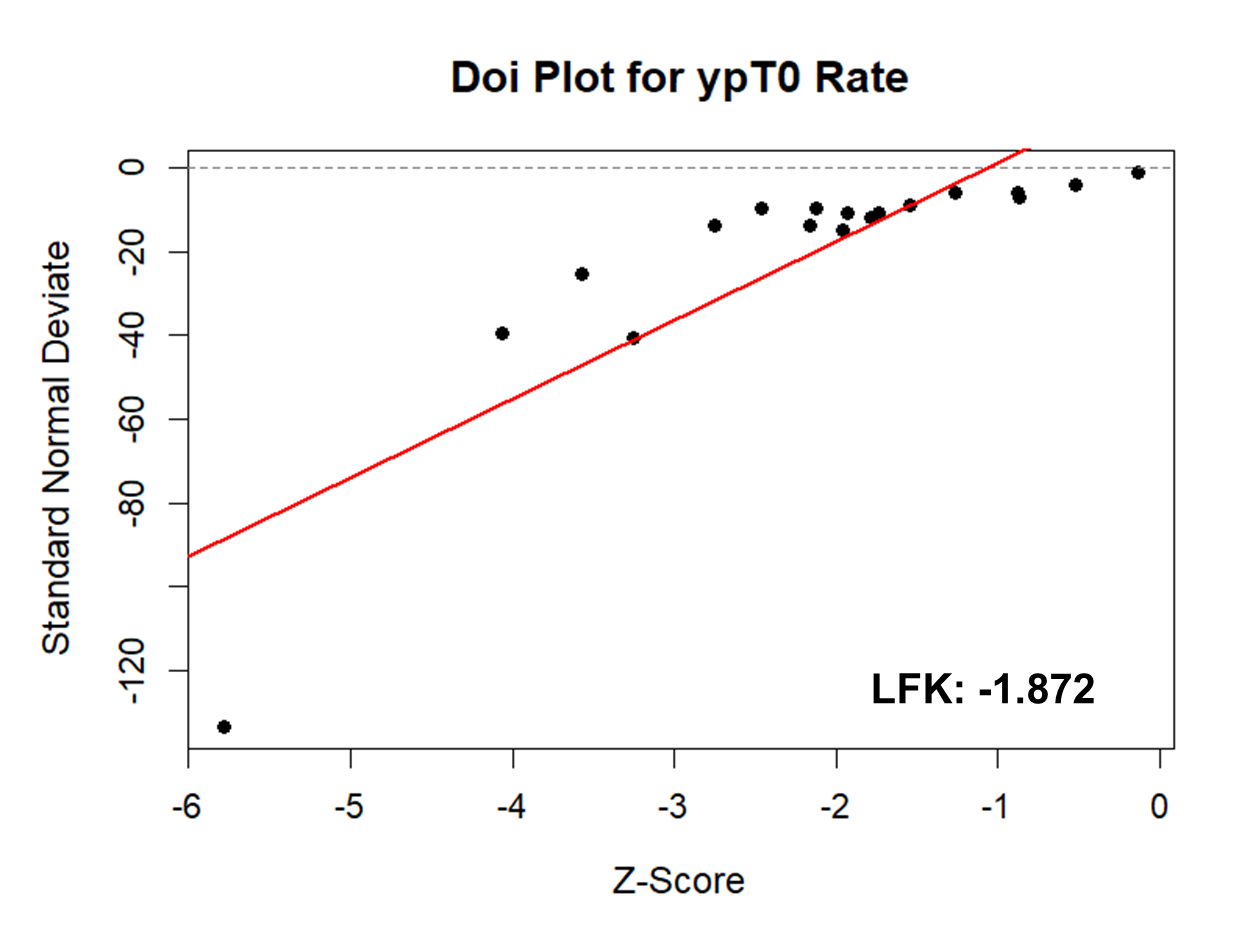


1. **eFigure 4:** Forest plot analysis of 2-year disease-free survival by treatment type
2. ICI


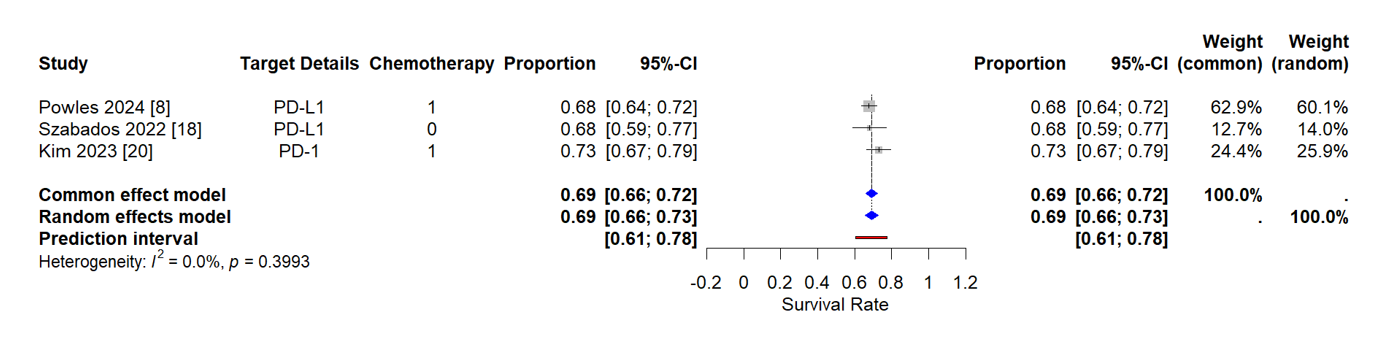


1. MTA


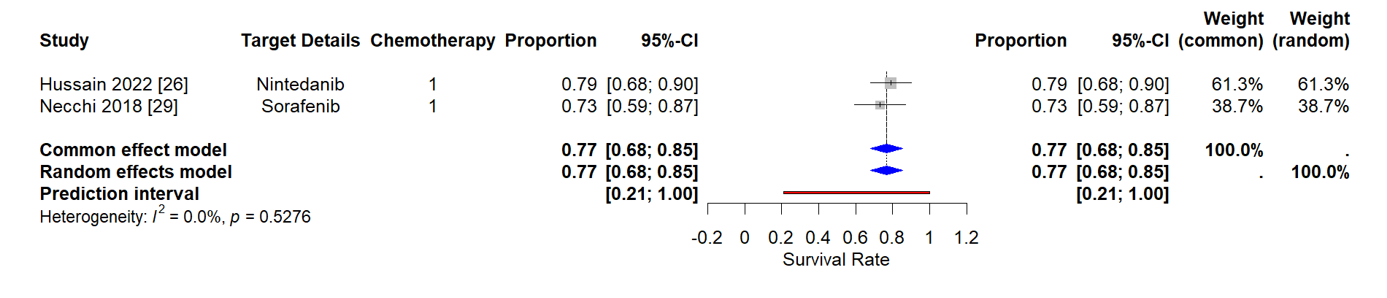


1. ADC


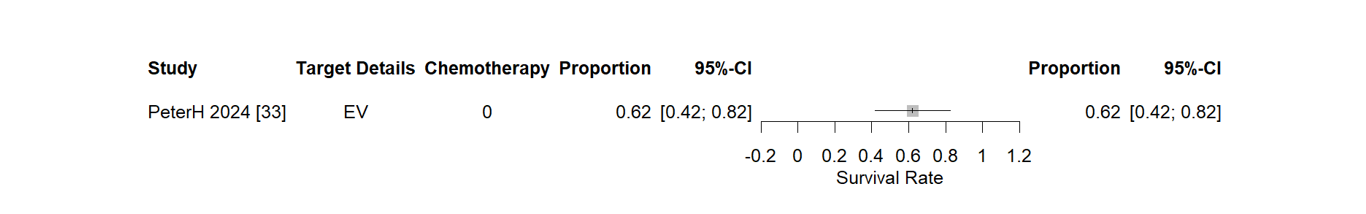


1. **eFigure 5:** Forest plot analysis of cystectomy rates by treatment type
2. ICI


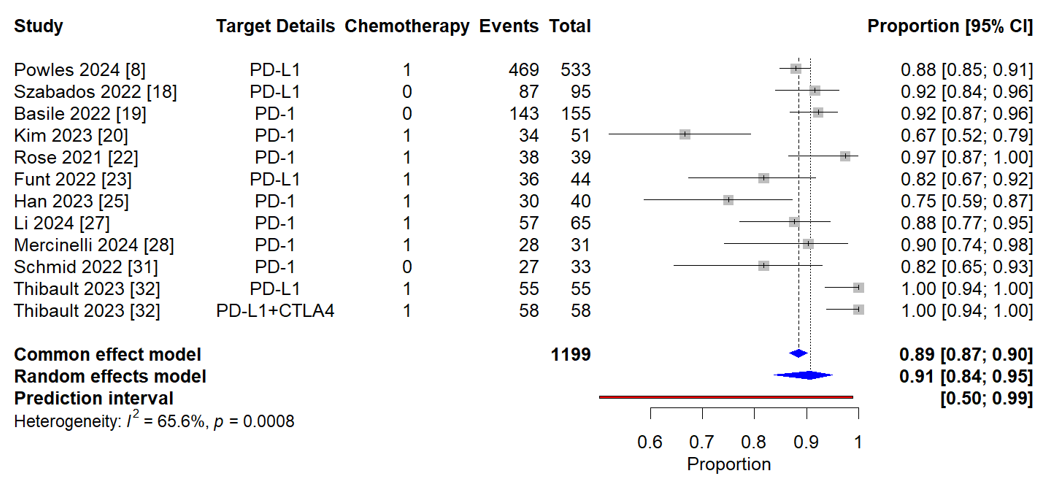


1. MTA


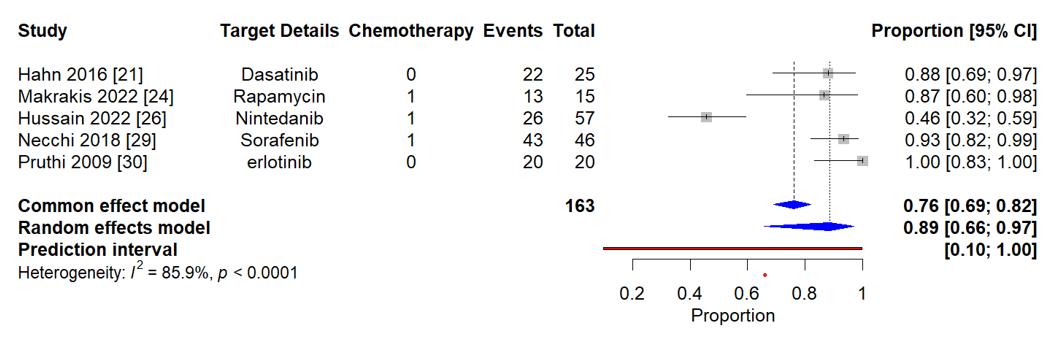


1. ADC


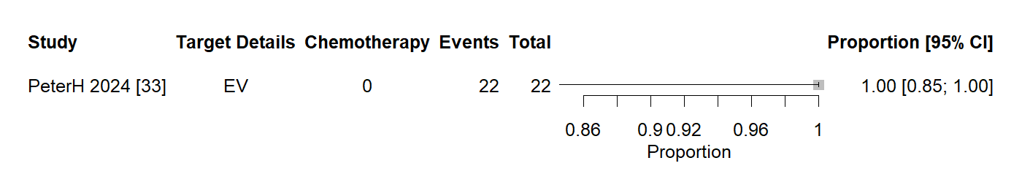


1. **eFIgure 6:** Forest plot analysis of R0 resection rates by treatment type
2. ICI


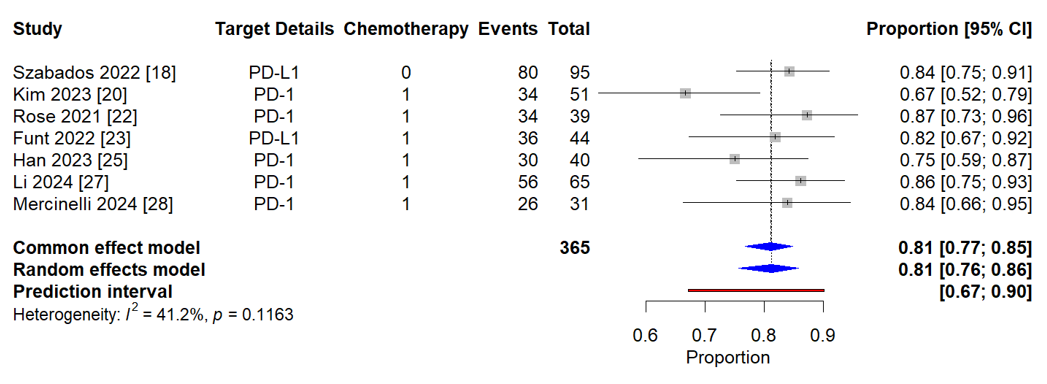


1. MTA


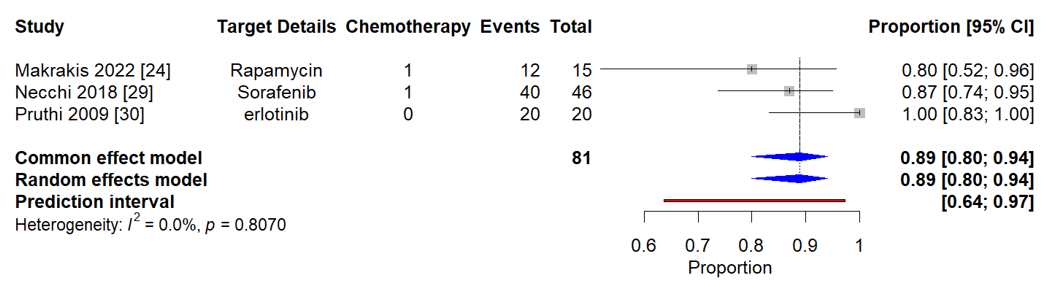


1. **eFigure 7:** Forest plot analysis of grade ≥3 adverse events during neoadjuvant therapy for MIBC
2. Anemia


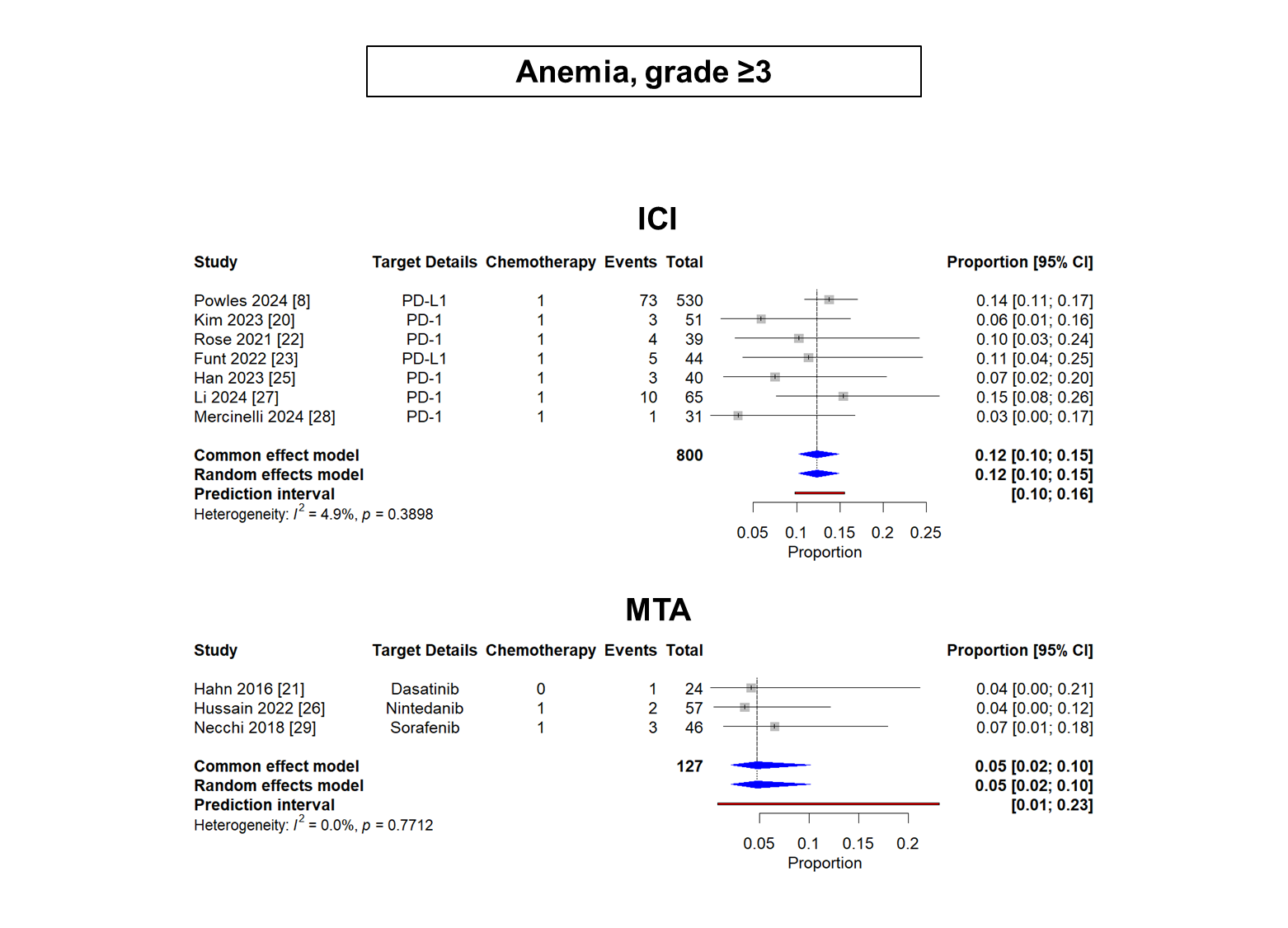


1. Constipation


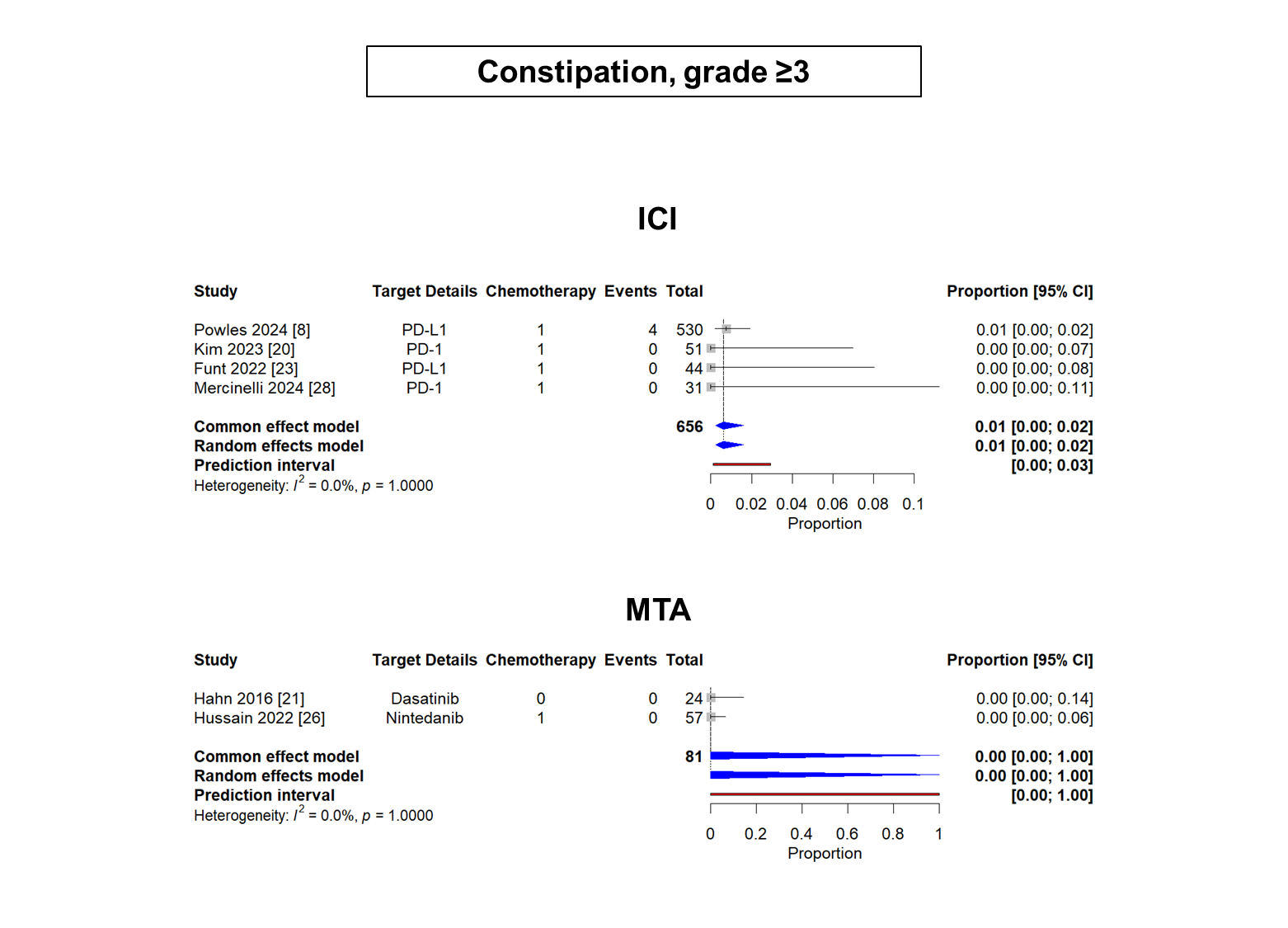


1. Diarrhea


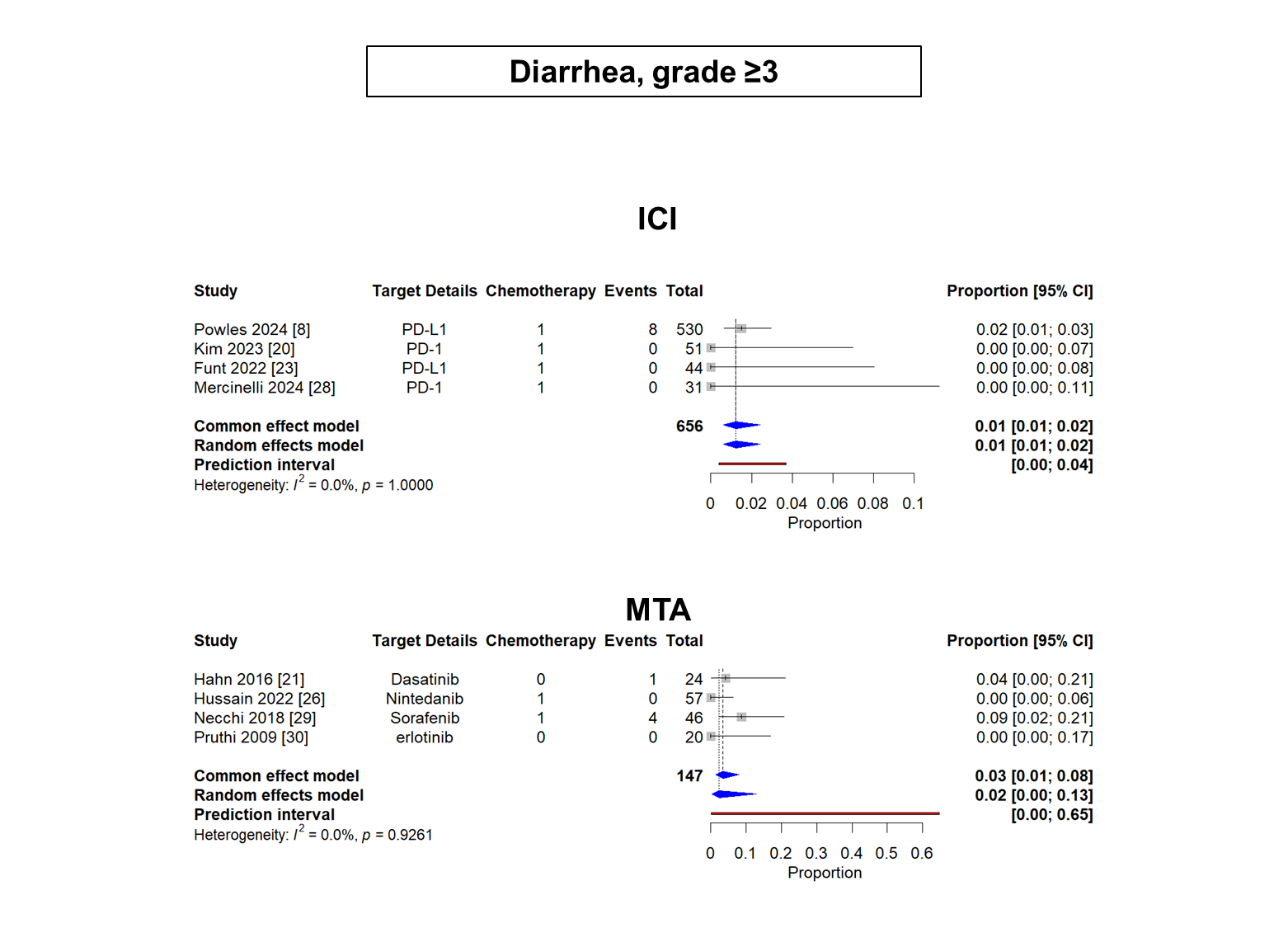


1. Fatigue


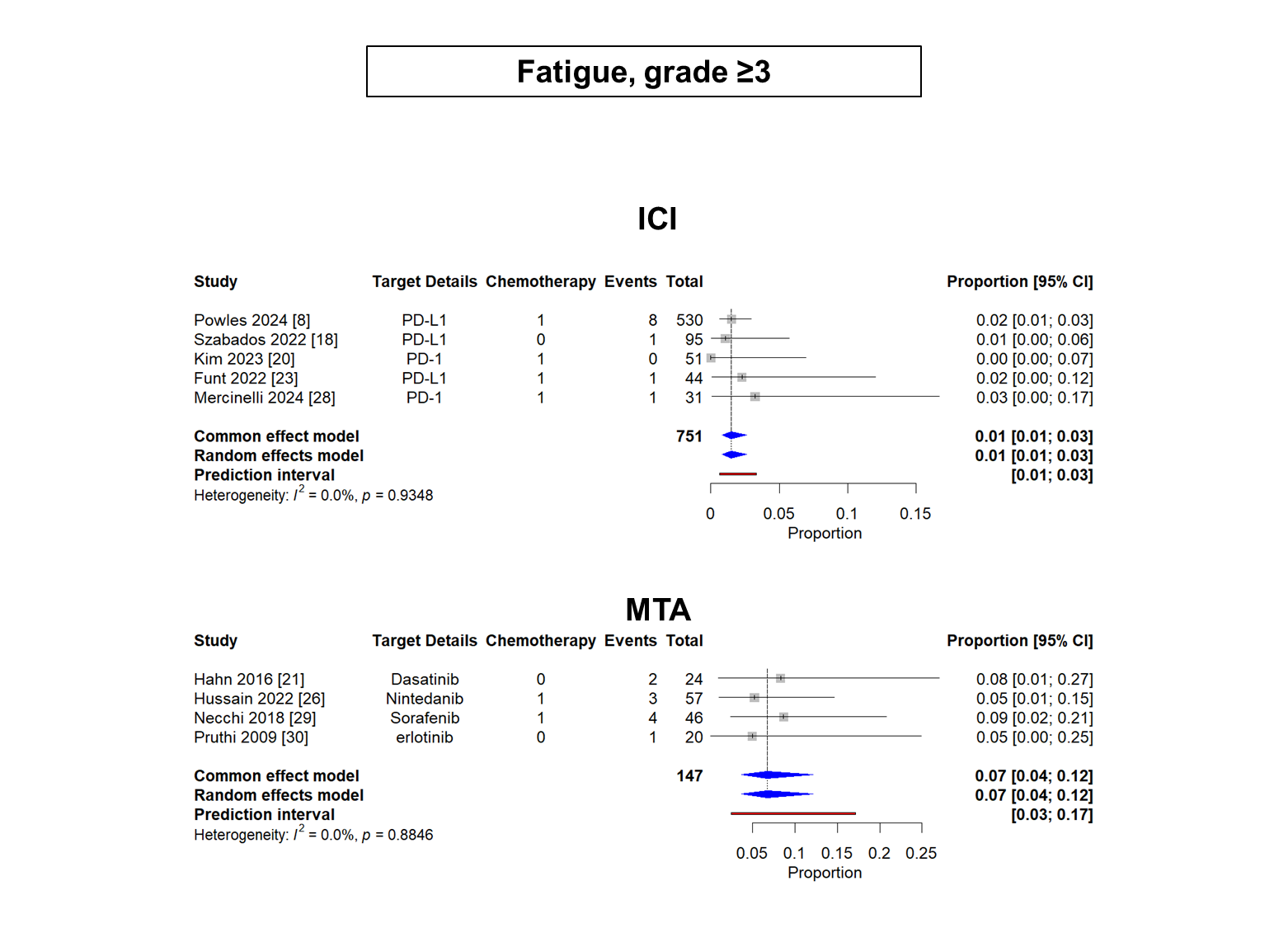


1. Nausea


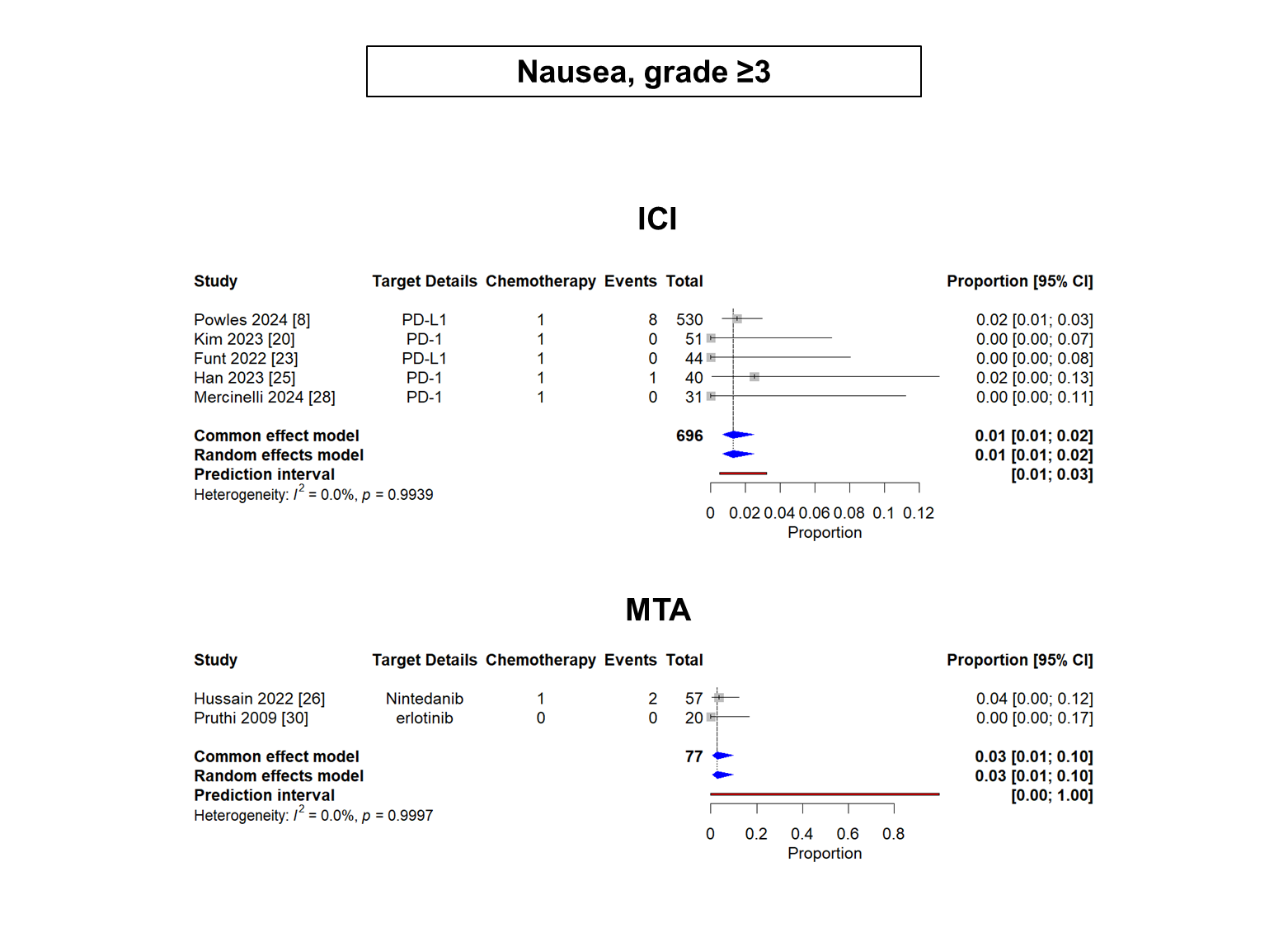


1. Neutropenia


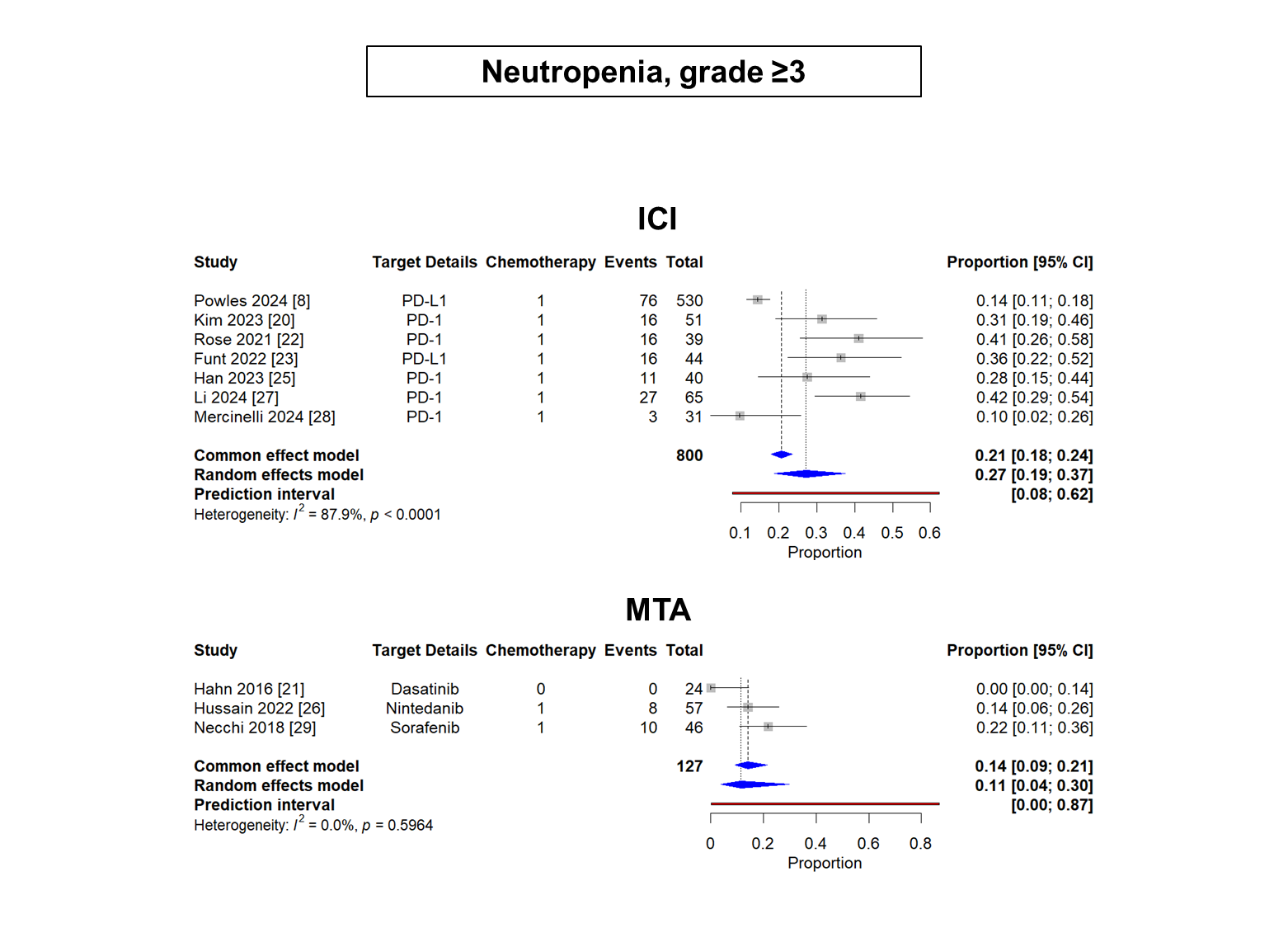


1. Thrombocytopenia


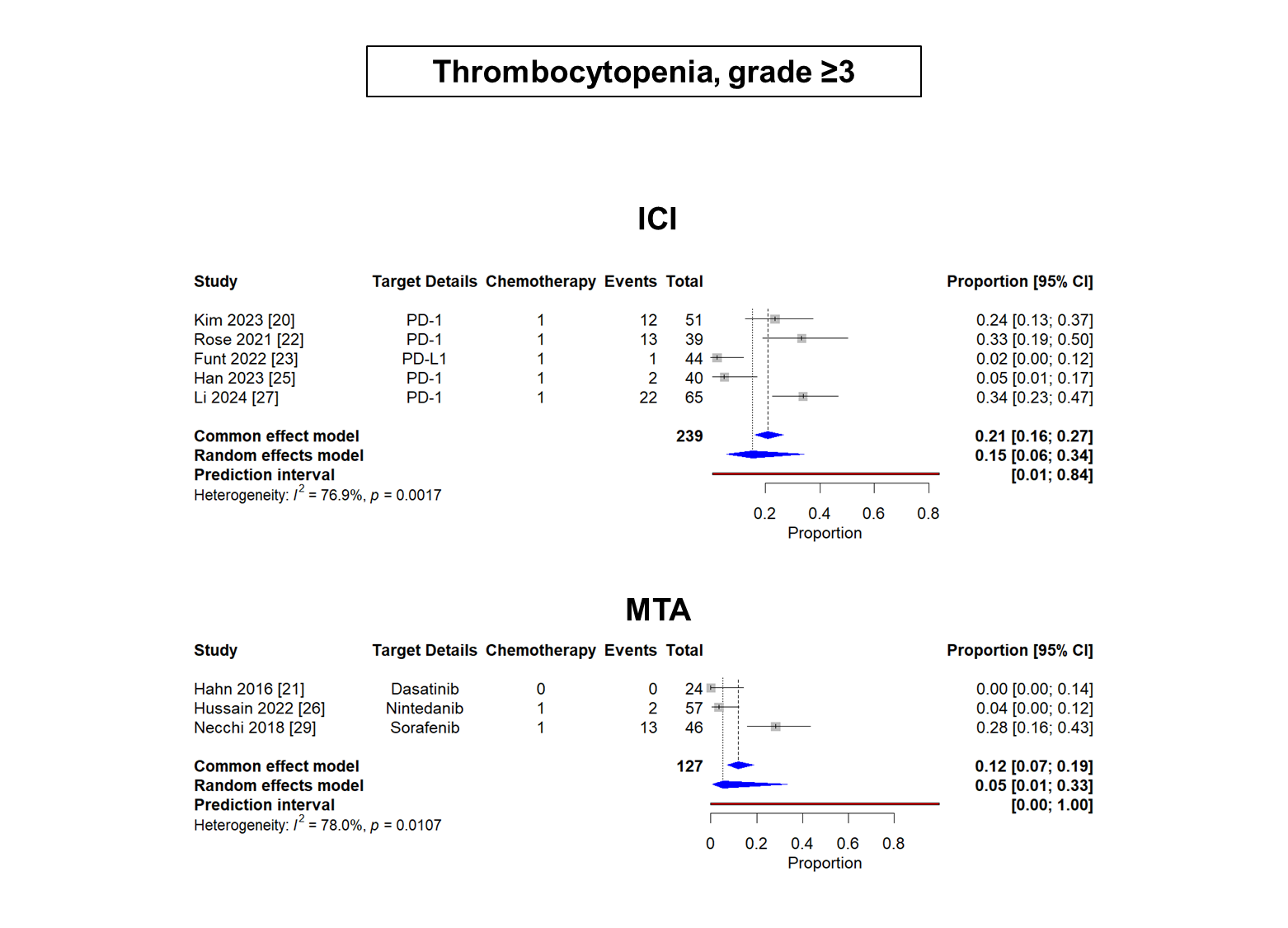


1. **eFigure 8:** Sensitivity analysis of pathologic response using radical cystectomy patients as denominator: (A) pathologic complete response rates and (B) downstaging rates
2. pathologic complete response rates


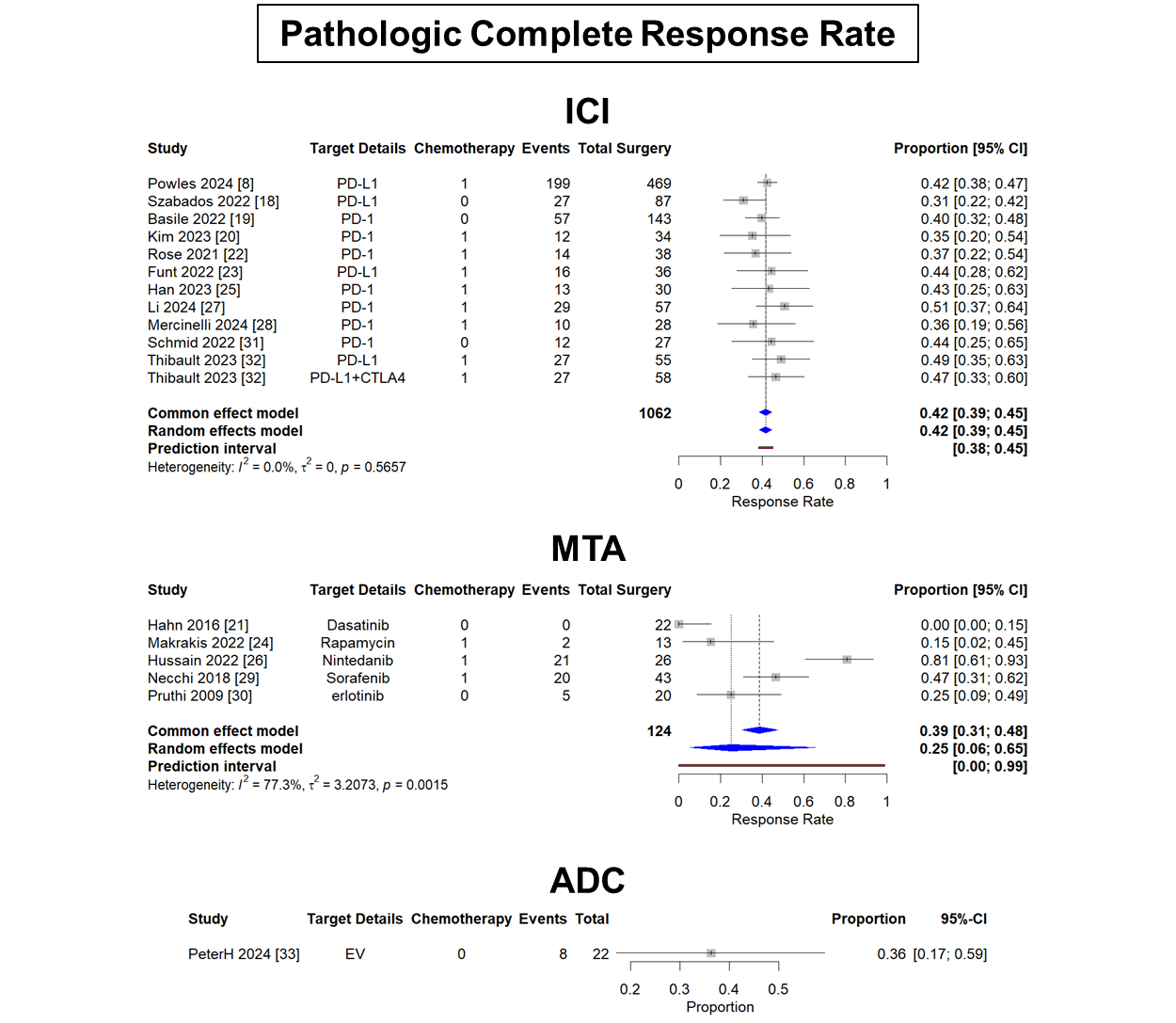


1. downstaging rates


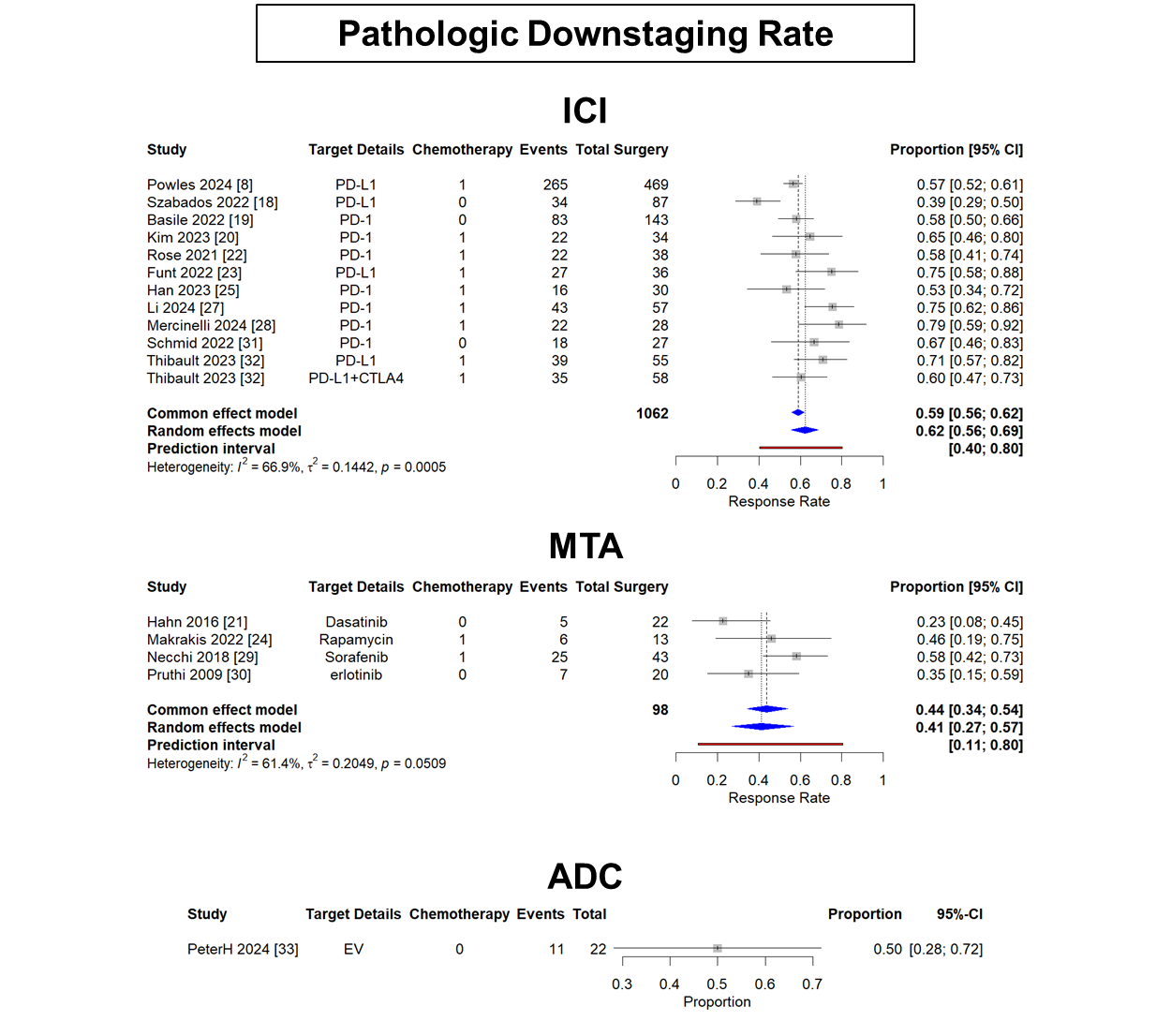

Supplement: Supplementary file 1 — Table S1: PRISMA 2020 checklist. Figure S1: PRISMA flow diagram of study selection. Table S2: Excluded studies with detailed justifications from meta‐analysis. Table S3: Baseline characteristics, treatment details, and safety data from clinical trials of novel neoadjuvant therapy in MIBC. Table S4: Comparative analysis of treatment‐related Adverse Events by therapeutic class. Figure S2: Risk of bias assessment of the included studies using RoB2 for randomized trials (A) and ROBINS‐I for non‐randomized studies (B). Figure S3: Doi Plot analysis for pathological complete response (ypT0) rate. Figure S4: Forest plot analysis of 2‐year disease‐free survival by treatment type. Figure S5: Forest plot analysis of cystectomy rates by treatment type. Figure S6: Forest plot analysis of R0 resection rates by treatment type. Figure S7: Forest plot analysis of grade ≥3 adverse events during neoadjuvant therapy for MIBC. Figure S8: Sensitivity analysis of pathologic response using radical cystectomy patients as denominator: (A) pathologic complete response rates and (B) downstaging rates. [file BCO2-6-e70031-s001.docx]
